# Evaluating the Neuroprotective and Acetylcholinesterase Inhibitory Properties of Four Calcineurin Inhibitor Drugs: Tacrolimus, Pimecrolimus, Cyclosporin A, and Voclosporin

**DOI:** 10.1007/s12035-025-05149-0

**Published:** 2025-09-15

**Authors:** Fatma Gonca Kocanci, Hamiyet Eciroglu Sarban, Fatma Yildiz

**Affiliations:** Department of Medical Laboratory Techniques, Alanya Alaaddin Keykubat University, Vocational High School of Health Services, Alanya, Antalya, Türkiye

**Keywords:** Acetylcholinesterase (AChE), Calcineurin inhibitor drugs, Neuroprotection, Oxidative stress, Molecular docking

## Abstract

Neurodegenerative diseases (ND), marked by progressive neuronal degeneration, often involve dysregulation of acetylcholinesterase (AChE), a key enzyme in cholinergic neurotransmission. AChE inhibition is a well-established therapeutic strategy for Alzheimer’s disease (AD), the most prevalent ND, as it aims to restore impaired cholinergic function. However, the effects of calcineurin inhibitors (CNIs), primarily used as immunosuppressants, on AChE activity remain largely unexplored. Recent evidence suggests CNIs possess neuroprotective properties, highlighting their potential for ND treatment. This study evaluated the binding affinities of FDA-approved CNIs—Tacrolimus (Tac), Pimecrolimus (Pim), Cyclosporine A (Csa), and Voclosporin (Voc)—to AChE via molecular docking and molecular dynamic simulation. AChE inhibition was assessed in vitro using the Ellman method and in H_2_O_2_-induced degenerative neuron-like SH-SY5Y cells via ELISA and qRT-PCR. Neuroprotection was examined through MTT assays and neurite analysis. Additionally, the antiapoptotic effect was examined by ELISA analysis measuring caspase-3. Docking studies confirmed strong AChE binding for all CNIs, with Voc exhibiting the highest affinity. Voc demonstrated superior in vitro AChE inhibition, surpassing galantamine at low concentrations. Cellular assays showed that CNIs, particularly Voc, significantly inhibited AChE expression at the gene level. Moreover, Voc markedly restored cell viability and reduced neuronal degeneration in H_2_O_2_-treated cells. These findings suggest CNIs, especially Voc, as promising candidates for ND treatment, targeting AChE overactivity and oxidative stress.

**Graphical Abstract:**

Created in BioRender. Hamiyet Eciroglu Sarban, (2025) https://BioRender.com/q77r137

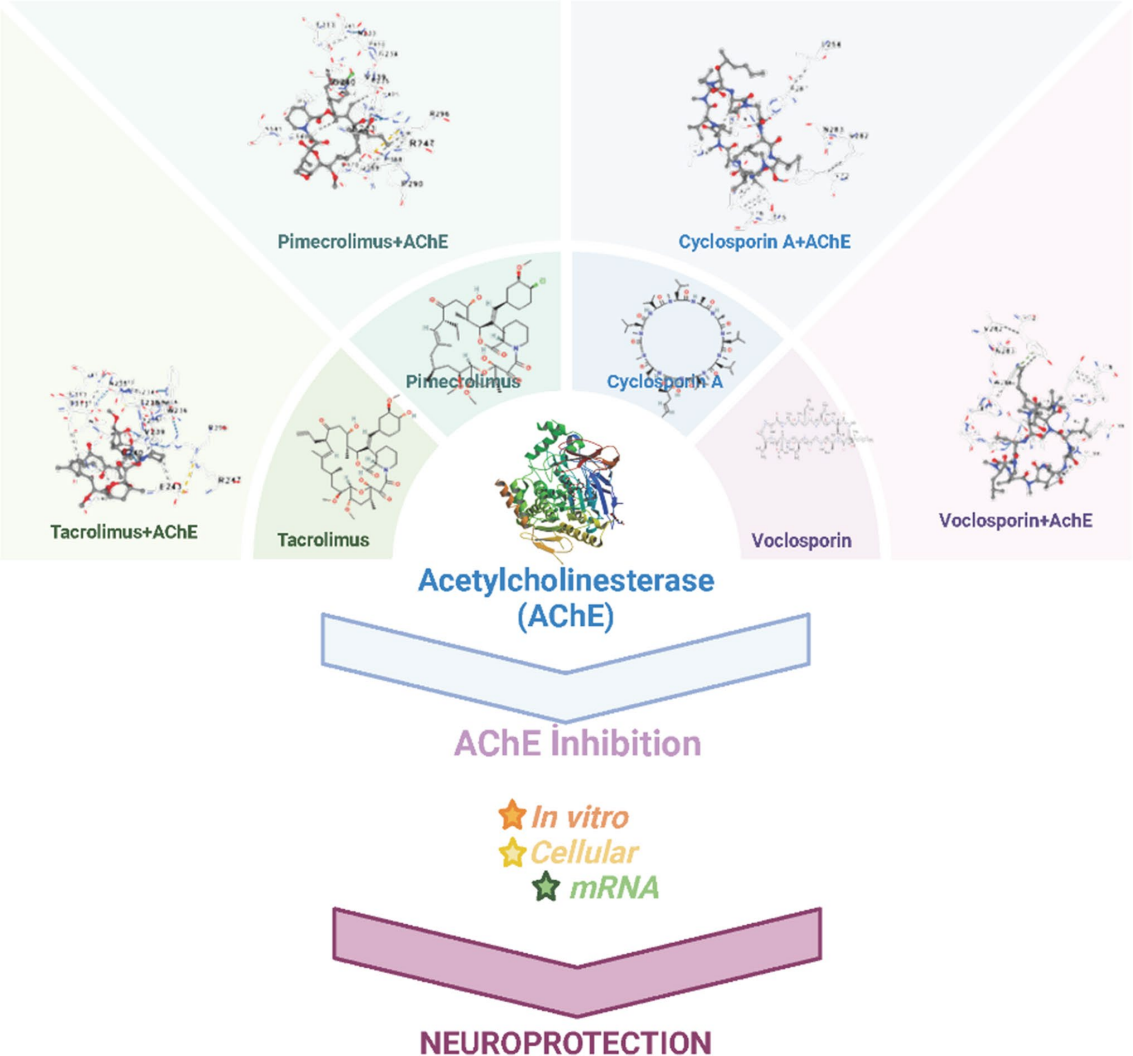

**Supplementary Information:**

The online version contains supplementary material available at 10.1007/s12035-025-05149-0.

## Introduction

NDs represent a significant global Health Challenge, with approximately 50 million people currently affected by dementia—a number expected to triple by 2050 [1]. AD, which constitutes the majority of dementia cases, is characterized by progressive cognitive decline, including memory loss, confusion, and an inability to perform daily activities, ultimately leading to loss of independence [2]. Pathologically, AD is marked by inflammation, the accumulation of amyloid plaques in the extracellular matrix, neurofibrillary tangles in the intracellular space, and an increase in intracellular AChE levels within neuronal cells [3–5]. The primary function of AChE is to rapidly terminate neural impulses by hydrolyzing acetylcholine (ACh), a neurotransmitter derived from acetyl coenzyme A, a byproduct of cellular respiration in mitochondria released into the synaptic cleft, while choline plays a crucial role in lipid metabolism [6].

Early stages of AD are associated with neuronal loss linked to increased AChE activity, leading to the premature hydrolysis of ACh before it can facilitate cholinergic transmission, contributing to cognitive decline. This observation led to the “cholinergic hypothesis,” which suggests that the cognitive decline in AD patients is primarily due to the loss of cholinergic function [7]. Based on this hypothesis, FDA-approved cholinesterase inhibitors (ChEIs) like donepezil, rivastigmine, and galantamine have been developed as the main therapeutic approach for managing mild to moderate AD [8]. These inhibitors prevent the hydrolysis of ACh by inhibiting AChE, thereby ensuring proper neural impulse conduction and slowing cognitive decline. However, while these ChEIs offer symptomatic relief, they do not halt disease progression and are often associated with adverse drug reactions. Consequently, searching for next-generation ChEIs that can provide similar efficacy with fewer side effects remains ongoing [9–12].

The “calcium hypothesis” has been proposed as another factor contributing to neuronal dysfunction and signal pathway disruptions in NDs, suggesting that calcium dysregulation may play a critical role [13]. Expanding on this, O’Day and Myre introduced the “calmodulin hypothesis,” positing that amyloid plaque production in AD could be regulated by a series of calcium-binding proteins [14]. Hyperactivation of calcineurin, a Ca^2+^/calmodulin-dependent phosphatase, has been observed in various AD models, where abnormal calcium signaling disrupts the interaction between calcineurin and calmodulin, leading to increased neuronal death [15–17]. Studies on both sporadic and familial AD have confirmed calcineurin hyperactivation as a central factor in AD pathogenesis [18, 19]. CNIs, by binding to the calcineurin-Ca^2+^-calmodulin complex, suppress calcineurin activation and can modulate pro-inflammatory cytokine expression, offering potential neuroprotective benefits. Research has shown that CNIs can restore memory function in animal models [20] and reduce the incidence of dementia in AD patients compared to the general population [21], suggesting they may help stabilize neuronal function in AD.

Furthermore, accumulating evidence suggests that CNIs may also possess direct neuroprotective effects independent of their immunosuppressive activity. Our recent studies demonstrated that CNIs such as Pim and Tac protect neuron-like SH-SY5Y cells against oxidative and inflammatory insults. Specifically, Pim was shown to attenuate H_2_O_2_-induced apoptosis, improve neuronal viability, and modulate key apoptotic pathways in differentiated SH-SY5Y cells [22]. In a separate study, it was demonstrated that Pim counteracts the pro-apoptotic and pro-oxidant effects of the microglial-derived secretome and oxidative stress, highlighting its dual anti-inflammatory and antioxidant potential [23]. Additionally, Tac exhibited significant anti-inflammatory and antioxidant actions in human microglial HMC3 cells, further supporting the role of CNIs in regulating microglial activation and neuroinflammation [24]. It has also been proven that Csa-loaded nanocarrier systems show protective effects against oxidative damage in neuron-like cells [25]. These findings underscore the multifaceted neuroprotective capacity of CNIs in both neuronal and glial models of oxidative stress.

Beyond our findings, a growing body of literature reveals that CNIs exert neuroprotection through a range of complementary and interconnected mechanisms. These include the inhibition of apoptosis, characterized by reduced DNA fragmentation, stabilization of the mitochondrial membrane potential, and prevention of cell death. CNIs are known to inhibit calcineurin activation, thereby blocking downstream pro-apoptotic signaling [26, 27]. Mitochondrial protection is another important aspect; CNIs prevent mitochondrial permeability transition during hypoxic or ischemic events and preserve mitochondrial morphology [28, 29]. CNIs also suppress neuroinflammation by inhibiting the calcineurin/NFAT pathway, leading to downregulation of pro-inflammatory cytokines such as TNF-α and IL-1β, reduced glial activation, and alleviation of amyloid beta (Aβ) toxicity [30–32]. Moreover, CNIs contribute to neuroprotection by reducing nitric oxide production and limiting ROS accumulation, both of which are major contributors to oxidative neuronal injury [33, 34]. Their ability to modulate calcium homeostasis is critical for preserving neuronal function, particularly in models of α-synuclein toxicity, where Tac reduces sustained cytosolic calcium elevations and prevents downstream calcineurin overactivation [35, 36]. Recent evidence also shows that CNIs can enhance DNA integrity by preventing Aβ-induced strand breakage [37] and promote synaptic plasticity, essential for neurite regeneration and synaptic stability [38]. In the context of excitotoxicity, CNIs have been shown to inhibit NMDA receptor-mediated neuronal injury [39].

Despite these multifaceted and well-documented neuroprotective actions, the effects of CNIs on AChE inhibition remain insufficiently characterized. While previous studies have focused on inflammation, apoptosis, and oxidative damage, no comprehensive comparative study has yet evaluated multiple CNIs in terms of their direct impact on AChE activity and expression under a unified experimental model. This represents a significant gap in the literature, particularly given the central role of AChE in cholinergic dysfunction associated with AD and related ND.

Drug repurposing, which identifies new uses for existing drugs beyond their original indications, offers a strategic approach to address this gap. It leverages the existing pharmacological and safety data of approved or clinically staged drugs, thus expediting development and reducing cost and risk [40, 41]. One commonly used method in drug repurposing is computational 3D molecular docking, which predicts interactions between biological macromolecules and various ligands through non-covalent bonding to form specific complexes. This method allows for the prediction of interaction energies and binding affinities between a target and ligand, providing insights into molecular interactions before conducting in vivo and in vitro studies.

In this study, we present the first comparative investigation of four FDA-approved CNIs—Tac, Pim, Csa, and Voc—evaluating their AChE inhibitory potential as well as their neuroprotective effects under oxidative stress conditions. The in vitro AChE inhibition profiles were assessed via Ellman’s assay, and their efficacy against H_2_O_2_-induced degeneration in differentiated SH-SY5Y cells was examined through ELISA, qRT-PCR, MTT assays, and neurite analysis. The novel aspect of this study lies in its integrative approach: combining in silico docking, biochemical inhibition analysis, gene expression, and functional cellular assays in a unified experimental model to explore both cholinergic modulation and oxidative stress protection. This comprehensive evaluation provides new insights into the potential repurposing of CNIs for ND treatment.

## Materials and Methods

### Molecular Docking

The binding affinities between the ligands Tac, Pim, Csa, Voc, and Gal with the target protein AChE were assessed through a molecular docking study utilizing CB-Dock2 software (Version 2) (available at: https://cadd.labshare.cn/cb-dock2/php/index.php) [42]. CB-Dock2 is integrated with AutoDock Vina, enabling a comprehensive analysis of the interactions between the protein and the ligands, which includes the identification of binding pocket sites, molecular docking, and evaluation of docking scores. The crystal structure of the AChE (PDB ID: 7E3D) was retrieved from the RCSB Protein Data Bank (https://www.rcsb.org/), while the ligands employed in this docking investigation were sourced from the PubChem database (https://pubchem.ncbi.nlm.nih.gov/). The apo-human AChE structure represents the most recent and updated conformation of the enzyme, offering improved resolution and accuracy in the depiction of the enzyme’s active site and binding pockets. PDB ID: 7E3D includes updated crystallographic data and refined model parameters, including Rwork and Rfree values of 0.2029 and 0.2417, respectively [43]. These values indicate a more accurate and reliable structural model, which is crucial for detailed docking studies where precision in ligand binding predictions is essential. The updated structural information allows for better assessment of the interactions between AChE and its inhibitors, contributing to more accurate predictions of binding affinities.

### Molecular Dynamics Simulations

The conformational flexibility of AChE in complex with various Ligands was analyzed using the CABSflex 2.0 server (available at https://biocomp.chem.uw.edu.pl/CABSflex2). CABSflex utilizes a coarse-grained Monte Carlo simulation framework to model residue-level fluctuations in protein structures under near-physiological conditions.

The apo-human structure of human AChE was used as the receptor model for all simulations. Ligand-bound complexes were generated using previously obtained docking poses. The ligands included in the simulation were Tac, Pim, Csa, Voc, and Gal, all of which were previously docked to AChE using the CB-Dock2 platform.

Each complex structure was submitted individually to the CABSflex 2.0 server. Default parameters were used, including the number of simulation cycles (50), the temperature value (1.4), and distance restraints derived from the input structure. The main output consisted of Root Mean Square Fluctuation (RMSF) profiles for each residue, indicating the predicted flexibility within the protein backbone. These results were used to assess the structural stability of the receptor in the presence of different ligands.

Hydrogen bond interactions between AChE and the tested ligands were identified and analyzed using *BIOVIA Discovery Studio Visualizer* (Dassault Systèmes, San Diego, CA, USA). The docked complexes were imported into the software, and hydrogen bonds were characterized based on default geometric criteria (distance ≤ 3.5 Å and donor–acceptor angle ≥ 120°). Key hydrogen bond parameters including bond distance, donor and acceptor atoms, and angular configurations were recorded. The results were tabulated to compare the binding interactions of each ligand with the AChE active site, providing insights into the potential stability and specificity of the ligand–receptor complexes.

### Computational ADMET Predictions

The ADMET properties of the selected compounds were predicted using a combination of in silico tools. The chemical structures of the compounds were retrieved from PubChem (https://pubchem.ncbi.nlm.nih.gov/) and subjected to in silico screening for ADMET profiling using the ADMETlab 2.0 tool (http://admet.scbdd.com/) [44]. This platform provides a comprehensive prediction of ADMET properties, including molecular weight, number of hydrogen bond acceptors and donors, topological polar surface area (TPSA), LogP, acute toxicity, genotoxic and non-genotoxic carcinogenicity, mutagenicity, blood–brain barrier (BBB) penetration, plasma protein binding, and volume of distribution, among other essential parameters.

### In Vitro Acetylcholinesterase (AChE) Inhibition Assay and IC _50_Determination 

The inhibition of AChE activity in vitro was evaluated following the methodology outlined by Ellman et al. [45], with some modifications implemented. Various concentrations (0.01–10 μM) of Tac (FK506) (10007965, Cayman Chemical Company), Pim (SML1437; SIGMA ALDRICH), Csa (B1922 Apexbio), and Voc (FV28719-Biosynth) were tested. Gal (G1660; SIGMA ALDRICH) was included in the assays as a positive control due to its well-established AChE inhibitory activity. The enzyme activity was expressed as a percentage relative to a control assay conducted without any inhibitor. The percentage inhibition of AChE activity was determined by comparing the absorbance values with those of the negative control, using the following equation:

AChE inhibition % = ((A_0_ − A_1_)/A_0_) × 100.

In this equation, A_0_ represents the absorbance of the control lacking the sample, while A_1_ indicates the absorbance of the sample tested.

The half-maximal inhibitory concentration (IC_50_) values of CINs and Gal against AChE were calculated using nonlinear regression analysis. Dose–response curves were generated and analyzed using GraphPad Prism version 8.0, applying a sigmoidal dose–response model with variable slope (four-parameter logistic regression). IC_50_ values were derived from the fitted curves and are expressed in micromolar (µM).

### Cell Culture and Treatment

The SH-SY5Y(CRL-2266) human neuroblastoma cell line used in this study was acquired from the SAP Institute (Ankara, Türkiye). Cells were cultured under standard conditions and used for experiments between passages 5 and 20 to maintain phenotypic consistency and avoid passage-related variability. To ensure the validity of the experimental data and avoid confounding effects, the cell line was routinely tested for Mycoplasma contamination using a PCR-based Mycoplasma detection kit (MycoFluor™ Mycoplasma Detection Kit, Thermo Fisher Scientific). All cultures tested negative for Mycoplasma prior to use in any experimental procedure. SH-SY5Y cells were cultured in DMEM/F12 medium (11,320,074, Gibco, Thermo Fisher, USA) supplemented with 10% (v/v) heat-inactivated fetal bovine serum (FBS) (BI04-007-1A; Bio. Ind) and a penicillin–streptomycin mixture (1:100; P0100-790; Cegrogen). All cultures were incubated in a CO_2_ incubator at 37 °C with 5% CO_2_ (v/v) and a humidity of 95%. Differentiated cells were plated at 1.0 × 10^4^ cell/cm^2^ confluency and treated with retinoic acid (RA) (R2625-50MG, Sigma Aldrich) at a 10 µM concentration in DMEM/F12 1% FBS for 6 days with RA pulses on every other day. The (d)*-*SH-SY5Y cells with acquired neuronal properties were used in our experiments involving toxin application and drug treatments.

### Cell Viability Assay

(d)-SH-SY5Y cells were cultured in collagen-coated 96-well plates (1.0 × 10^4^ cell/well). Cell cultures were co-treated with H_2_O_2_ (250 µM) and various concentrations (0.01–10 µM) of Tac, Pim, Csa, and Voc for 24 h; then, the cell viability rates of all groups were investigated using thiazolyl blue tetrazolium bromide (MTT) (146,345- abcam) as previously described [23]. The appropriate dose and time for H_2_O_2_ used in our study were determined according to the findings obtained in a preliminary study [23]. Data are expressed as a percentage of cell viability compared to control cultures.

### Assessment of Caspase-3 Levels

The quantification of cleaved caspase-3 levels in cell lysates was performed using a commercially available human cleaved caspase-3 ELISA kit (E6970HU, BT Lab), according to the manufacturer’s instructions. SH-SY5Y cells were seeded in collagen-coated 6-well plates (1.0 × 10^5^ cell/well) and differentiated. Cells were exposed to 250 μM H_2_O_2_ in the presence or absence of CNI compounds for 24 h. Following treatment, cells were collected, diluted with PBS, and destroyed by multiple freeze–thaw cycles, both treated and untreated. The levels of cleaved caspase-3 in the cell lysates were measured in the order recommended by the manufacturer. The BCA assay (44,132, Expedeon) was used to compare the total protein contents in treated and untreated cells.

### Microscopic Examination of (d)-SH-SY5Y Cells

SH-SY5Y cells were cultivated on collagen-coated 6-well plates at a density of 1.0 × 10^5^ cells/well. Following the differentiation and treatments, microscopic examination of the cell groups was assessed using an inverted microscope (Zeiss Axio) at × 20 objective. Images were taken with multiple independent images for an individual treatment. Images were analyzed for neurite lengths and the number of homogeneous neurites per cell by NeuronJ of ImageJ software; soma size was determined by the Neurphology plugin of ImageJ [46]. Cells with cell extensions longer than twice the cell body diameter were considered neurite-bearing cells. 100 cells were counted from 10 different areas selected to determine neurite density (number of neurites per cell) and neurite length for each sample. The data were expressed as the mean ± SEM from three independent experiments.

### Cellular AChE Activity

SH-SY5Y cells were cultivated on collagen-coated 6-well plates at a density of 1.0 × 10^5^ cells/well. Following the differentiation of the cells, they were co-treated with H_2_O_2_ at a concentration of 250 μM and drugs at 0.1 μM for a duration of 24 h. To extract the total protein from the treated (d)-SH-SY5Y cells, the cultures were rinsed twice with cold phosphate-buffered saline (PBS), followed by scraping to collect the cells. The resulting cell suspension was transferred to 1.5 mL tubes, which were then centrifuged at 10,000 × g for 5 min at 4 °C. The supernatant was carefully discarded, and the cell pellets were lysed using RIPA buffer. The total protein concentration present in the extracts was quantified using the Bicinchoninic Acid (BCA) assay [47]. Any remaining protein was stored at − 80 °C until the time of enzymatic analysis. Relative AChE activity was determined using an equal amount of total protein and employing an Enzyme-Linked Immunosorbent Assay (ELISA) (Human AchE (Acetylcholinesterase) Kit, ELK4614-96 T), following the manufacturer’s guidelines.

#### mRNA Expression Analysis of AChE by QRT-PCR

SH-SY5Y cells were cultivated on collagen-coated 6-well plates at a density of 1.0 × 10^5^ cells/well. Following the differentiation of the cells, they were co-treated with H_2_O_2_ at a concentration of 250 μM and drugs at 0.1 μM for a duration of 24 h. Following the cell treatments, total RNA was extracted from the cells using the EZ-10 Spin Column Total RNA Miniprep Kit (BS136, Biobasic Canada) in accordance with the manufacturer’s instructions. The purity and concentration of the isolated RNA were assessed by measuring absorbance at 260 nm and 280 nm. cDNA was synthesized from 1 μg of total RNA using the OneScript Plus cDNA Synthesis Kit (G236, Abmgood). Subsequent to cDNA synthesis, quantitative real-time PCR (qRT-PCR) was conducted on a Roche Light Cycler 96 real-time PCR System utilizing the SYBR Green PCR Kit BlasTaq 2X qPCR MasterMix (G891, Abmgood) according to established protocols. All amplification reactions for each sample were performed in triplicate, and the experiments were repeated at least three times. The relative mRNA expression levels were quantified using the 2^−ΔΔCt^ method, normalizing the data against glyceraldehyde-3-phosphate dehydrogenase (GAPDH). Primer sequences are listed as follows: GAPDH forward, 5′-ACAACTTTGGTATCGTGGAAGG-3′ and reverse, 5′-GCCATCACGCCACAGTTTC-3′; AChE forward, 5′-AGCAGTACGTTAGTCTGGACCT-3′ and reverse, 5′-TGCTTGCTGTAGTGGTCGAA-3′.

#### Statistics

All data were presented as the mean ± S.E.M. The Shapiro–Wilk test was used for the normality of the distribution. Group comparisons were performed using one-way analysis of variance (ANOVA) followed by post hoc Tukey’s HSD (for parametric data with equal variance)/Games-Howell post hoc test (for parametric data with unequal variance). Data analysis and graphical presentations were performed using GraphPad Prism 8.0 software (USA). In all analyses, *p* values equal to or below 0.05 were considered statistically significant.

## Results

### Molecular Docking

Molecular docking was conducted to evaluate the binding capacity and interaction modes between the ligands Tac, Pim, Csa, and Voc with AChE, using galantamine as a positive control. The ligands are represented using a ball-and-stick model. The Vina scores obtained from the docking simulations indicate that lower scores correlate with enhanced stability of the binding interactions, signifying stronger ligand–protein interactions. The molecular docking results demonstrated that all tested CNI drugs (Tac, Pim, Csa, and Voc) successfully interacted with AChE at the active site. Voc exhibited the strongest binding affinity (− 11.6 kcal/mol), followed by Tac (− 9.1 kcal/mol), Pim (− 8.5 kcal/mol), and Csa (− 7.3 kcal/mol). These findings suggest that Voc’s superior binding is likely due to its optimized interaction with the catalytic triad residues, enhancing its inhibitory potential compared to galantamine (− 8.3 kcal/mol). Detailed binding interactions are summarized in Table 1.

**Table 1:**
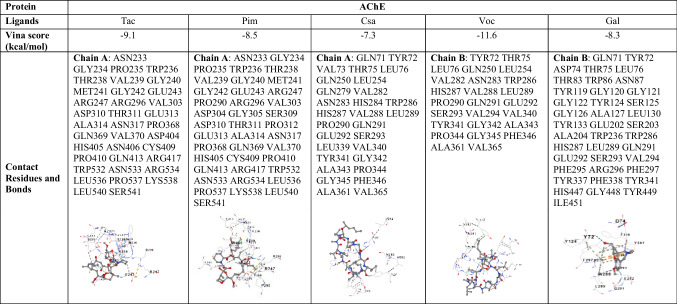
Molecular docking results of AChE and ligands

### RMSF Analysis

Root-mean-square fluctuation (RMSF) analysis provided insights into the dynamic stability of the AChE–ligand complexes (Supplementary Data 1). The Tac-bound AChE structure exhibited RMSF values predominantly between 1.6 and 2.0 Å, indicating moderate flexibility in the binding region while preserving the enzyme’s overall structural integrity. In contrast, the Pim-bound complex demonstrated lower RMSF values, ranging from 1.2 to 1.7 Å around the active site, suggesting a highly stable binding conformation. The AChE–Csa complex displayed the highest degree of local flexibility, with RMSF values between 1.8 and 2.5 Å, reflecting a dynamic interaction pattern despite its strong binding affinity. Voc binding resulted in the most rigid complex, with remarkably low RMSF values (1.0 to 1.4 Å) at TRP236 and surrounding residues, supporting the presence of a structurally stable interaction. Finally, the Gal–AChE complex showed RMSF values ranging from 1.4 to 1.8 Å, indicative of a generally stable binding interface. Overall, the RMSF data emphasize the differential dynamic behavior of each ligand, with Pim and Voc showing enhanced structural stabilization of the AChE active site.

### Hydrogen Bond Interaction Analysis

To further elucidate the molecular interactions between AChE and the tested ligands, hydrogen bond analyses were performed. As summarized in Supplementary Data 2 and Supplementary Data 3, all ligands exhibited hydrogen bonding with key amino acid residues within or near the AChE active site.

Voc formed the highest number of hydrogen bonds (10 total), including interactions with residues such as GLU313, ASN533, and PRO537. These bonds displayed donor–acceptor distances below 3.2 Å, with favorable bond angles, suggesting strong and specific interactions that support the docking-based predictions of high binding affinity. Pim also formed multiple hydrogen bonds with residues including SER203 and TYR341, with acceptable geometric parameters, indicating moderately strong interactions. In contrast, Tac exhibited weaker binding geometry, with suboptimal bond angles (e.g., < 40°), which may reflect a less stable or non-optimal orientation within the binding pocket. Csa formed fewer but geometrically favorable hydrogen bonds. Notably, Gal formed stable interactions with TRP286 and GLN291, both of which are critical residues involved in ligand stabilization within the catalytic gorge of AChE.

These findings are in agreement with the molecular docking results and provide further structural evidence supporting the differential binding affinity and potential inhibitory mechanisms of the tested CNIs and reference compound Gal.

### ADMET Profiling of the Calcineurin İnhibitors

To evaluate the pharmacokinetic properties and safety profiles of the studied CNIs in the context of their potential neuroprotective applications, ADMET analyses were conducted and summarized in Table 2.

**Table 2:** The ADMET analysis of the four calcineurin inhibitors

**Drug name**	**Molecular Weight**	**Number of hydrogen bond** **acceptors**	**Number of hydrogen bond** **donors**	**Total polar surface area** (**TPSA)**	**LogP**	**Acute Toxicity**	**Non-genotoxic carcinogenicity**	**Genotoxic carcinogenicity** **Mutagenicity**	**Blood–brain barrier (BBB)** **Penetration**	**Plasma protein binding (PPB) (%)**	**Volume of distribution (Vd)**
Tac	803.480	13	3	178.360	5.120	0.000	0.000	0.000	-	82.662	1.353
Pim	809.450	12	2	158.130	6.079	0.000	2	3	-	83.386	1.762
Csa	1201.840	23	5	278.800	4.731	0.000	0.000	0.000	-	82.732	0.868
Voc	1213.840	23	5	278.800	4.350	0.000	0.000	0.000	-	83.662	0.887

All four compounds exhibit high molecular weights, with Csa and Voc being the Heaviest at approximately 1200 Da, whereas Tac and Pim are slightly Lighter, around 800 Da. The number of hydrogen bond acceptors and donors varies, with Tac and Pim having fewer acceptors (13 and 12, respectively) compared to Csa and Voc (23 each). The total polar surface area (TPSA) is similarly higher for Csa and Voc (~ 278 Å^2^) than for Tac (178 Å^2^) and Pim (158 Å^2^), indicating differences in their potential for cell permeability and solubility.

Lipophilicity, indicated by LogP values, is notably higher for Pim (6.079) compared to the other compounds. This suggests enhanced lipid membrane affinity, which may influence bioavailability and distribution. None of the tested drugs demonstrated acute toxicity, genotoxic carcinogenicity, or mutagenicity, but Pim exhibited a non-genotoxic carcinogenicity score, indicating potential risks that warrant further investigation.

Plasma protein binding (PPB) was uniformly high for all compounds, exceeding 82%, reflecting a strong affinity for serum proteins. The volume of distribution (Vd) varied across the drugs, with Tac and Pim showing higher values (1.353 and 1.762, respectively) compared to Csa and Voc (0.868 and 0.887, respectively).

These findings underline the distinct pharmacokinetic and safety profiles of the studied CNIs, providing valuable insights into their potential therapeutic applications and limitations.

### In Vitro AChE Inhibition

**Fig. 1:**
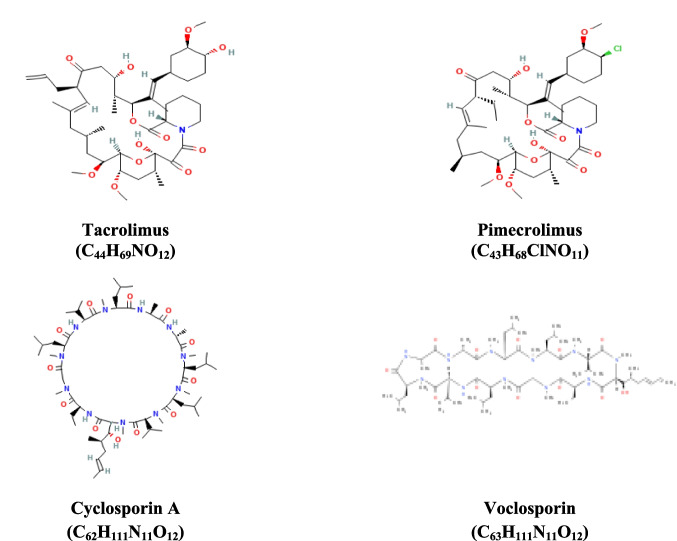
Chemical structures and molecular formulas of the studied compounds

**Fig. 2:**
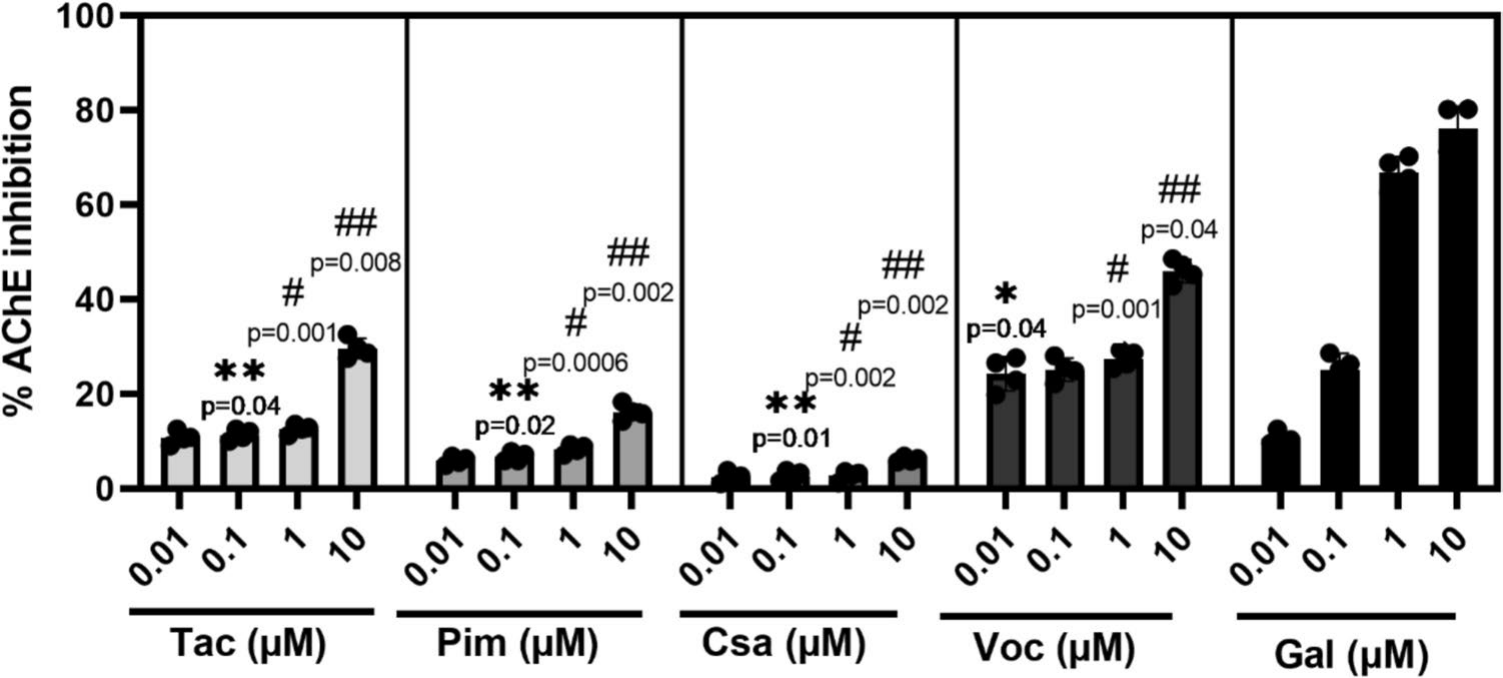
In vitro inhibitory effects of Tacrolimus (Tac), Pimecrolimus (Pim), Cyclosporin A (Csa), and Voclosporin (Voc) on recombinant human acetylcholinesterase (AChE). The drugs were tested at concentrations ranging from 0.01 to 10 µM, and the level of enzymatic inhibition was quantified. Galantamine (Gal) was used as a positive control. *n* = 4. (*p < 0.05 as compared to the 0.01 µM Gal; **p < 0.05 as compared to the 0.1 µM Gal; ^#^*p* < 0.05 as compared to the 1 µM Gal; ^##^*p* < 0.05 as compared to the 10 µM Gal). (F: Fisher’s test statistic, DFn: Degrees of Freedom numerator (between-group degrees of freedom) and DFd: Degrees of Freedom denominator (within-group degrees of freedom) values were as: 685.9, 4, and 15)

The drugs Tac, Pim, Csa, and Voc (Fig. 1) were incubated with recombinant human AChE and the level of enzymatic inhibition quantified. The drugs Tac, Pim, Csa, and Voc (Fig. 1) were incubated with recombinant human AChE and the level of enzymatic inhibition quantified. Galantamine was utilized as a positive control. These results showed that the potency of the drugs to inhibit AChE was in the order: Voc˃Tac˃Pim˃Csa. Voc was more potent an inhibitor of human AChE enzyme. At a concentration of 0.01 µM Voc exhibited a higher AChE inhibitory effect compared to galantamine. At a concentration of 0.1 µM, the AChE inhibitory effects of Voc were similar to that of galantamine. However, at concentrations of 1 and 10 µM, none of the drugs demonstrated efficacy comparable to that of galantamine in inhibiting AChE (Fig. 2). To quantitatively assess the inhibitory potency of the CNIs on AChE, enzyme kinetics analysis was performed based on dose–response curves. The half-maximal inhibitory concentration (IC_50_) values were calculated for CNIs. The resulting IC_50_ values were as follows: Tac—4.243 ± 0.1 µM, Pim—2.148 ± 0.1 µM, Csa—2.335 ± 0.2 µM, Voc—0.004 ± 0.0002 µM, and Gal—0.212 ± 0.01 µM. Among the tested compounds, Voc exhibited the strongest inhibitory effect on AChE, with an IC_50_ significantly lower than that of Gal (Data not shown).

###  Effects of H_2_O_2_ and/or Calcineurin Inhibitors on (d)-SH-SY5Y Cell Viability, Neurite Outgrowth, and Apoptotic Activity (Caspase-3 Levels)

**Fig. 3:**
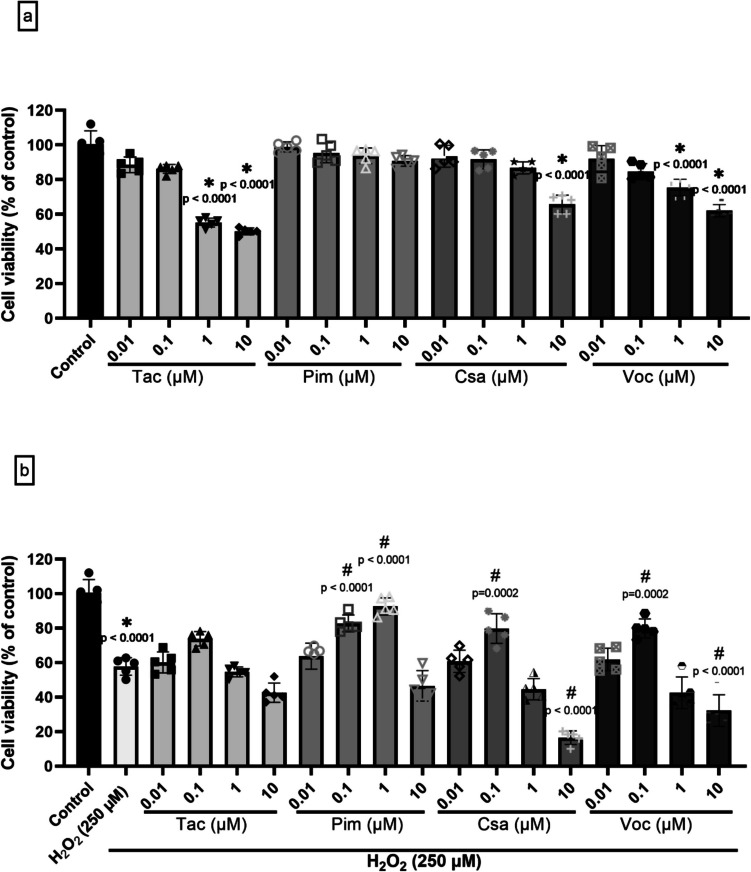

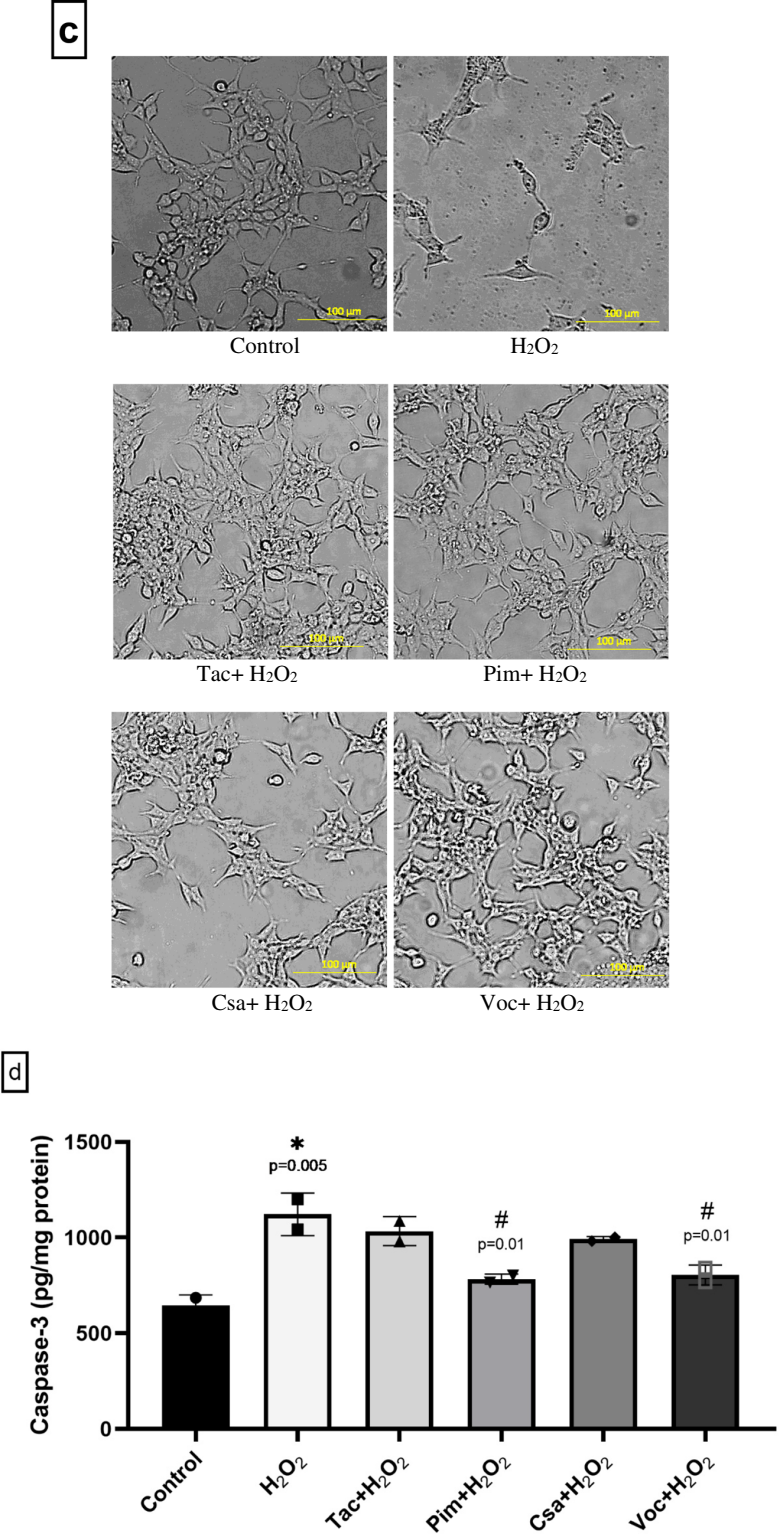
Effects of calcineurin inhibitors on (d)-SH-SY5Y cell viability, neurite outgrowth, and apoptotic activity. (d)*-*SY-SY5Y cells were treated with various concentrations (0.01–10 μM) of Tacrolimus (Tac), Pimecrolimus (Pim), Cyclosporin A (Csa), or Voclosporin (Voc) (*n* = 5) (**a**) or with H_2_O_2_ (250 μM) and various concentrations (0.01–10 μM) of these drugs (*n* = 5) (**b**). Representative microscopic images illustrating morphological changes in cells treated with H_2_O_2_ alone and co-treated with H_2_O_2_ and 0.1 µM of each drug. Scale bars indicate magnification (**c**). Caspase-3 levels in (d)-SH-SY5Y cells treated with H_2_O_2_ and/or calcineurin inhibitors (**d**) (*n* = 2). *p < 0.05 as compared to the untreated cells as control, and.^#^*p* < 0.05 as compared to H_2_O_2_ treatment alone. F, DFn, and DFd values were as follows: 52.03, 16, 68 (**a**); 54.35, 17, 72 (**b**); 15.77, 5, 6 (**d**)

As shown in Fig. 3a, treatment with Tac, Csa, and Voc alone at concentrations up to 1 μM showed no cytotoxic effect in (d)-SH-SY5Y cells. In particular, Tac and Voc showed significant cytotoxicity at 1 and 10 μM (55.2 ± 2.5 75.5 ± 4.8, respectively at 1 μM and 50.1 ± 2.0 and 62.1 ± 3.6, respectively at 10 μM %cell viability) Similarly, Csa showed statistically significant cytotoxicity at 10 μM (65.9 ± 5.2%cell viability). In contrast, Pim did not show detectable cytotoxicity at any tested concentration (0.01–10 μM). These results indicate that all CNIs were well tolerated by (d)-SH-SY5Y cells at or below 1 μM, and 0.1 μM was chosen as the optimum concentration for subsequent co-treatment experiments.

In order to assess the protective effects of these drugs against H_2_O_2_-induced toxicity, (d)-SH-SY5Y cells were simultaneously treated with 250 μM H_2_O_2_ and various concentrations of the drugs. The results depicted in Fig. 3b demonstrate that exposure of (d)-SH-SY5Y cells to 250 µM H_2_O_2_ for 24 h resulted in a significant decrease in average cell viability, measuring at 57.8 ± 5% compared to the control group. This concentration was determined to be suitable for inducing neurotoxicity in subsequent experiments, supporting the conclusions drawn from our earlier study on the toxic effects of H_2_O_2_ in (d)-SH-SY5Y cells [23]. However, co-treatment with H_2_O_2_ (250 µM) and the drugs revealed concentration-dependent neuroprotective effects. Among the tested concentrations, 0.1 μM of Csa and Voc yielded the most consistent and statistically significant protective effects, significantly increasing cell viability compared to the H_2_O_2_-only group. Although Tac showed a slight numerical increase in viability at 0.1 μM, this effect was not statistically significant. In Pim, 1 µM concentration was the concentration that best protected cell viability against H_2_O_2_ toxicity, consistent with our previous findings [23] (Fig. 3b). Higher concentrations (1 and 10 µM for Tac, Csa, and Voc, 10 µM for Pim) failed to maintain this protective effect, potentially due to compound-induced cellular stress. Additionally, treatment with 10 μM Csa or Voc, when combined with H_2_O_2_, resulted in a further decrease in cell viability compared to H_2_O_2_ alone. This suggests a potential synergistic cytotoxic interaction between these drugs and oxidative stress, possibly due to mitochondrial dysfunction or impaired cellular stress responses triggered by the combined exposure. These findings highlight that while CNIs can offer neuroprotective effects at lower concentrations, higher concentrations may exacerbate oxidative damage, underscoring the importance of dose selection in therapeutic applications. Consequently, to ensure consistency and comparability across all treatment groups, subsequent experiments were conducted using a concentration of 0.1 µM for all tested compounds.

Microscopic examination supported these findings, showing improved cell morphology in the presence of 0.1 µM drug treatments combined with H_2_O_2_ (Fig. 3c). Control cells displayed a healthy, neuron-like morphology characterized by extensive neurite outgrowth and well-defined cell bodies. Exposure to H_2_O_2_ caused a marked reduction in neurite length, cell shrinkage, and irregular cell shapes, indicative of oxidative stress-induced cytotoxicity. In contrast, co-treatment with H_2_O_2_ and the CNI drugs at 0.1 µM concentration notably preserved the morphological integrity of the cells. Among the tested compounds, Voc demonstrated the most prominent protective effect, with treated cells exhibiting neurite structures and cell body shapes comparable to the untreated control group.

Treatment of (d)-SH-SY5Y cells with H_2_O_2_ alone resulted in a significant increase in caspase-3 levels compared to untreated controls, indicating enhanced apoptotic activity (*p* < 0.05). Among the CNIs co-administered with H_2_O_2_, Pim and Voc significantly attenuated this increase, suggesting a potential protective effect against H_2_O_2_-induced apoptosis (*p* < 0.05). In contrast, Tac and Csa did not significantly modulate caspase-3 expression in H_2_O_2_-treated cells (Fig. 3d). These findings underline the ability of Pim and Voc to effectively mitigate oxidative damage and maintain neuronal morphology under stress conditions, further supporting their neuroprotective potential.

In our analysis of neurite morphology, exposure to 250 μM H_2_O_2_ led to a significant reduction in both the number and length of neurites in (d)-SH-SY5Y cells compared to the untreated control group. Specifically, H_2_O_2_ treatment decreased the neurite index from 1.3 ± 0.5 to 0.8 ± 0.2 and reduced average neurite length from 50.2 ± 4.4 µm to 39.6 ± 6.3 µm. Co-treatment with CNIs (0.1 μM) produced differential effects on morphological parameters. Voc co-treatment fully preserved the neurite index and significantly increased neurite length compared to the H_2_O_2_ group (*p* < 0.05), indicating a robust protective effect. CsA showed partial protection by significantly improving the neurite index, although it did not significantly affect neurite length. In contrast, Tac and Pim failed to produce any significant improvement in either neurite index or length compared to the H_2_O_2_-treated group, indicating a lack of protective effect on neurite morphology (Table 3). These findings demonstrate that only Voc provided substantial morphological neuroprotection under oxidative stress.

**Table 3:** Effects of calcineurin inhibitors on neurite outgrowth in d-SH-SY5Y cells

Groups	Neurite index (neurite/cell)	Neurite length (μm)
Control	1.3 ± 0.5	50.2 ± 4.4
H_2_O_2_ (250 µM)	0.8 ± 0.2*^p=0.001^	39.6 ± 6.3*^p=0.001^
Tac (0.1 µM) + H_2_O_2_ (250 µM)	1.2 ± 0.1	34.5 ± 3.5
Pim (0.1 µM) + H_2_O_2_ (250 µM)	1.1 ± 0.3	39.8 ± 5.7
Csa (0.1 µM) + H_2_O_2_ (250 µM)	1.2 ± 0.4^#*p*=0.02^	38.8 ± 3.1
Voc (0.1 µM) + H_2_O_2_ (250 µM)	1.3 ± 0.3^#*p*=0.02^	45.4 ± 5.5^#*p*=0.04^

*p < 0.05 vs. control group (untreated cells). ^#^*p* < 0.05 vs. H_2_O_2_ treated group. F, DFn, and DFd values were as follows: 3.94, 5, and 54 for neurite index; and 19.43, 5, and 54 for neurite length, respectively. *n* = 10

### Cellular AChE Inhibition Effects of Calcineurin Inhibitors

**Fig. 4:**
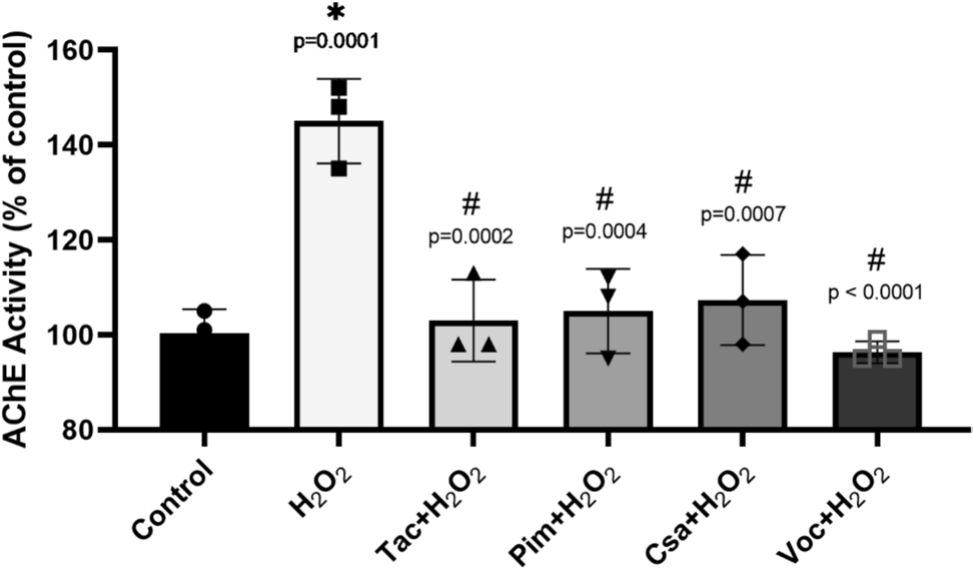
Effects of calcineurin inhibitors on cellular AChE activity in (d)-SH-SY5Y cells. Cells were treated with 250 µM H_2_O_2_ alone or co-treated with 250 µM H_2_O_2_ and 0.1 µM of Tacrolimus (Tac), Pimecrolimus (Pim), Cyclosporin A (Csa), or Voclosporin (Voc) for 24 hours. (*p < 0.05 as compared to the untreated cells as control, and.^#^*p* < 0.05 as compared to H_2_O_2_ treatment alone). (F, DFn, and DFd values were: 16.12, 5, 12). *n* = 3

In H_2_O_2_-treated cells, AChE activity increased significantly (1.45-fold, compared to the control), confirming its role in oxidative stress-induced neurodegeneration. Co-treatment with 0.1 μM of the tested drugs significantly reduced AChE activity in H_2_O_2_-treated cells. Among these, Voc exhibited a numerically greater reduction in AChE activity (96.3 ± 2.3%AChE activity); however, this difference did not reach statistical significance when compared to the other CNIs (Fig. 4).

### Effects of Calcineurin Inhibitors on the Expression of AChE-mRNA

**Fig. 5:**
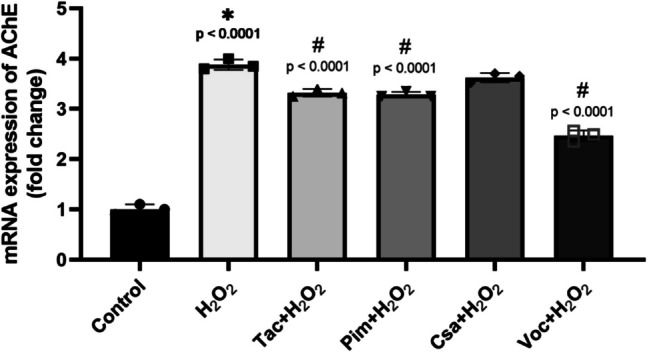
Effect of calcineurin inhibitors on AChE mRNA expression levels in (d)-SH-SY5Y cells treated with 250 µM H_2_O_2_ exhibited significantly elevated AChE mRNA levels, which were reduced upon co-treatment with 0.1 µM of Tacrolimus (Tac), Pimecrolimus (Pim), Cyclosporin A (Csa), or Voclosporin (Voc) for 24 h. (*p < 0.05 as compared to the untreated cells as control, and.^#^*p* < 0.05 as compared to H_2_O_2_ treatment alone). (F, DFn, and DFd values were: 405.8, 5, 12. *n* = 3

RT-qPCR analysis revealed that H_2_O_2_ exposure led to a significant upregulation of AChE mRNA expression. Co-treatment with the drugs at 0.1 µM significantly reduced AChE mRNA levels compared to the H_2_O_2_-treated group for Tac, Pim, and Voc. Voc demonstrated the most pronounced reduction. However, CsA did not cause a statistically significant decrease in AChE mRNA expression relative to H_2_O_2_ alone (Fig. 5). These results suggest that the tested CNIs may suppress AChE transcription under oxidative stress conditions.

## Discussion

The therapeutic landscape for AD has remained relatively static over the past two decades, predominantly relying on second-generation AChE inhibitors such as donepezil, rivastigmine, and galantamine, all of which were approved between 1997 and 2001. These AChEIs function by inhibiting AChE, thereby elevating ACh levels and mitigating the cholinergic deficits associated with AD [48].

Despite their clinical utility, existing AChEIs have limitations, including variable efficacy across patients and adverse side effects such as gastrointestinal disturbances and cardiovascular issues [49]. For instance, tacrine, the first AChEI approved in 1993, was withdrawn due to severe hepatotoxicity, highlighting the need for safer alternatives [50]. Consequently, there is a pressing need for the development of novel AChEIs that could offer enhanced efficacy, improved safety profiles, and better cost-effectiveness. Furthermore, emerging research into novel inhibitors may reveal new mechanisms of action or enhance understanding of cholinergic system modulation, potentially leading to more effective and personalized therapeutic strategies. Continued exploration in this field is essential for overcoming the limitations of current treatments and ultimately improving the management of AD.

The concept of “drug repurposing” involves evaluating existing medications, initially developed for one indication, for their potential efficacy in treating different diseases. This strategy leverages the well-established safety profiles and pharmacokinetic data of these drugs, reducing the time and costs associated with drug development. Repurposing can also reveal novel therapeutic applications that were not originally anticipated, thus accelerating the availability of new treatments [51]. In this context, the current study investigates the neuroprotective and AChEI effects of all FDA-approved CNI drugs (Tac, Pim, Csa, and Voc) as therapeutic agents for AD.

The selection of CNI drugs for this study was guided by their established mechanisms of action and emerging evidence linking calcineurin signaling to neurodegeneration [18, 52–54]. CNIs such as Tac, Pim, Csa, and Voc, initially developed for managing organ transplant rejection and autoimmune disorders, exhibit potent modulatory effects on calcium-dependent signaling pathways [55]. Dysregulation of calcium homeostasis is closely associated with the neuropathological features of AD, such as neuronal dysfunction, Aβ aggregation, and tau hyperphosphorylation [57–61]. By inhibiting calcineurin, these drugs can attenuate aberrant calcium signaling, potentially reducing the downstream neurotoxic effects that contribute to AD pathogenesis.

In addition to their impact on calcium homeostasis, CNIs possess anti-inflammatory properties, which are particularly relevant to AD, a condition characterized by chronic neuroinflammation [17]. Microglial activation, driven by pro-inflammatory cytokines, exacerbates neuronal damage in AD [62]. By suppressing calcineurin-dependent pathways, these drugs may reduce microglial overactivation and modulate the release of neuroinflammatory mediators, thereby preserving neuronal integrity [53]. These multifaceted mechanisms—modulating cholinergic dysfunction, calcium dysregulation, and neuroinflammation—highlight the potential of CNIs to offer a comprehensive therapeutic approach for AD. Furthermore, their well-characterized safety profiles and pharmacokinetics, established through decades of clinical use, provide a strong foundation for their repurposing as novel AD therapies.

In our study, the binding potential of Tac, Pim, Csa, and Voc to AChE was investigated by molecular docking. To further evaluate the suitability of these compounds for potential therapeutic use, ADMET analysis was performed. In addition, both in vitro AChE inhibitory effects of these drugs and cellular AChE inhibitory activities in neuron-like SH-SY5Y cells exposed to H_2_O_2_-induced oxidative stress were examined at the gene level. In addition, the effects of the drugs on cell viability and neurite lengths were determined in the same cell model. This evaluation aimed to determine the ability of these drugs to modulate AChE activity under oxidative conditions and their neuroprotective effects.

Tac, approved in 1994, is a potent immunosuppressant primarily used in organ transplantation [63]. Beyond its CNI effects, recent studies have highlighted its anti-inflammatory and antioxidative actions, suggesting potential neuroprotective benefits [64–68]. However, it is important to note that Tac is associated with nephrotoxicity and neurotoxicity, which limits its broader therapeutic use [69]. Tac, chemically classified as a macrolide, exerts its effects by binding to the immunophilin FKBP-12 (FK506-binding protein), forming a novel FKBP12-FK506 complex. This complex inhibits peptidyl-prolyl isomerase activity and suppresses calcineurin, a critical phosphatase involved in T-lymphocyte signaling. By inhibiting calcineurin, the FKBP12-FK506 complex disrupts the transcription of interleukin-2 (IL-2), thereby effectively attenuating T-cell activation and immune response [70]. Pim is utilized topically for atopic dermatitis and offers targeted immunosuppression with minimal systemic effects [71]. Emerging research indicates that Pim may also exert neuroprotective effects, potentially due to its ability to modulate inflammatory responses and oxidative stress in neural contexts [23, 72]. Like the Tac used, Pim binds to immunophilins to form a complex, and this complex inhibits the calcineurin phosphatase component. This mechanism suppresses cytokine production in T-lymphocytes and thus inflammatory stability. However, Pim was developed to have local rather than systemic effects and is usually used topically in the treatment of inflammatory skin diseases such as atopic dermatitis [73]. Csa, a foundational drug in transplantation since 1983, functions by binding to cyclophilin and inhibiting calcineurin, thereby suppressing T-cell activation [74]. While primarily known for its nephrotoxicity and hypertension, recent studies have begun to explore its neuroprotective potential, particularly through mechanisms involving modulation of neuroinflammation and protection against neurotoxic insults [75–78]. Voc, a newer drug approved in 2021, provides enhanced pharmacokinetics and a more favorable side effect profile compared to older CNIs [79, 80]. In addition to its primary role in transplantation, Voc may show promising results for neuroprotection by reducing neuroinflammation and oxidative damage, potentially due to its ability to modulate calcineurin activity more precisely. Collectively, while these drugs are well-established for their CNI roles, their emerging neuroprotective properties warrant further investigation in the context of NDs and cholinergic dysfunction.

The binding affinity of human AChE for its substrates and inhibitors is a key determinant of its physiological and pharmacological roles. The enzyme’s binding affinity is primarily dictated by its active site, which includes a catalytic triad composed of serine, histidine, and glutamate residues [81, 82]. This triad is essential for the hydrolysis of acetylcholine and interacts with the substrate through precise electrostatic and hydrophobic interactions.

Recent studies have elucidated that human AChE exhibits high specificity and affinity for ACh, facilitated by a deep and narrow active site gorge that effectively accommodates and catalyzes the breakdown of the neurotransmitter [83]. This structural feature also has implications for the binding of various inhibitors. Inhibitors that target the active site of the enzyme and can form strong non-covalent interactions with the catalytic triad and other important residues in the active site generally show high binding affinity [84]. In summary, the binding affinity characteristics of human AChE are fundamental to understanding its function and interaction with inhibitors. Continued research into these interactions is essential for advancing the development of more effective and selective therapies for NDs.

In this study, the SH-SY5Y cell line was selected as a model system to investigate the neuroprotective and AChE inhibitory effects of CNI drugs under oxidative stress conditions. SH-SY5Y cells, derived from a human neuroblastoma, are widely utilized in neurodegenerative research due to their ability to differentiate into neuron-like cells with morphological, biochemical, and functional characteristics of dopaminergic and cholinergic neurons [85]. This makes them a robust model for studying neuronal responses to various treatments and stressors. A significant advantage of SH-SY5Y cells is their ease of culture and differentiation, which enables the consistent generation of neuron-like cells for experimental studies [86]. (d)-SH-SY5Y cells express key neuronal markers, including neurofilament proteins and synaptic proteins, and exhibit functional ACh release and AChE activity, making them particularly suitable for exploring cholinergic dysfunction in diseases such as AD [86]. Moreover, SH-SY5Y cells are highly amenable to genetic and pharmacological manipulations, allowing for the evaluation of specific molecular pathways and drug effects in a controlled environment [87]. Their responsiveness to oxidative stress, induced by agents like H_2_O_2_, further strengthens their relevance for modeling neurodegenerative processes, including oxidative damage and its impact on neuronal survival and function [88]. Overall, the SH-SY5Y cell line offers a cost-effective, reproducible, and human-relevant model system for studying the cellular and molecular mechanisms underlying NDs and for evaluating the therapeutic potential of candidate drugs, including the immunosuppressive agents investigated in this study.

Our findings demonstrate that these CNI drugs exhibit varying degrees of AChE inhibitory activity, with Voc showing the most promising effects. Molecular docking studies revealed that Voc, in particular, has a high binding affinity to AChE, surpassing the standard AChE inhibitors, Gal. This superior binding interaction is attributed to optimized contact with the active site residues of AChE, which likely enhances its inhibitory potential. Although Tac, Pim, and Csa AChE binding affinities were less pronounced compared to Voc and galantamine, they still demonstrated potential as AChE inhibitors. These results suggest that these drugs, particularly Voc, may offer superior AChE inhibition due to their optimal interactions with the enzyme’s active site.

A comparative analysis of the molecular docking results reveals that Voc, Gal, Tac, Pim, and Csa interact with AChE at both overlapping and distinct binding sites, suggesting different modes of inhibition across these compounds. Gal primarily engages residues within the catalytic active site, including Ser203, His447, and Tyr337, and several residues in the anionic and oxyanion subsites. This is characteristic of classical AChE inhibitors designed to block acetylcholine hydrolysis directly. Gal’s interactions suggest a competitive inhibition mechanism, where it binds to the catalytic region, preventing the enzyme from efficiently hydrolyzing acetylcholine. Voc, in contrast, binds predominantly to residues located at the peripheral anionic site (PAS), such as Tyr72, Thr75, Leu76, Trp286, and His287, without direct interaction with the catalytic triad. This suggests that Voc may exert its inhibitory effect through allosteric modulation, potentially altering the enzyme’s conformation or interfering with substrate access. The broader and more hydrophobic interaction profile of Voc, involving residues like Val340 and Phe346, may provide greater binding stability despite the lack of direct catalytic site engagement. Its cyclic peptide structure, enhanced lipophilicity, and rigid conformation may contribute to more stable interactions at the PAS, reinforcing its potential as a non-competitive inhibitor. The distinct pharmacophoric features of Voc thus support both its binding advantage and therapeutic promise in modulating AChE in ND contexts. This non-competitive or mixed-type inhibition mechanism offers a novel approach to cholinergic modulation, especially in ND contexts. Tac, Pim, and Csa also interact with AChE, but their binding profiles differ significantly from those of Gal and Voc. Tac binds at several locations, including residues ASN233, GLY234, PRO235, TRP236, THR238, VAL239, and others, with no direct interaction with the catalytic triad. The broad range of interactions and inclusion of hydrophobic residues like Val303 and Phe346 may indicate an allosteric inhibition mechanism, similar to Voc. Pim shares a similar binding profile with Tac, with interactions at residues such as ASN233, GLY234, and TRP236, as well as additional residues like PRO290 and GLY305. The overlap with Tac in binding regions suggests that Pim may also act through an allosteric modulation mechanism, influencing AChE activity indirectly without direct catalytic site engagement. Csa, while also binding to AChE, interacts with a more distinct set of residues, including GLN71, TYR72, VAL73, THR75, LEU76, and others. These interactions are similar to those observed in Voc at the PAS, but Csa’s binding profile is more limited, suggesting that its inhibitory mechanism may be less potent or less specific compared to the other compounds discussed here. In summary, while Gal acts as a direct competitive inhibitor through interactions with the catalytic site, Voc, Tac, and Pim are more likely to exert their effects through allosteric inhibition, with a broader range of binding sites. Csa also shows an allosteric inhibition profile but with a more limited binding interaction. These differences in binding mechanisms suggest that each molecule may offer unique therapeutic potential for modulating AChE activity in ND, with non-competitive inhibitors like Voc potentially providing novel avenues for cholinergic modulation.

In addition to the molecular docking studies, which revealed favorable binding affinities of Tac, Pim, Csa, and Voc to AChE, MD simulations provided deeper insight into the conformational stability and flexibility of these ligand–AChE complexes. RMSF analysis demonstrated that the AChE–Voc complex exhibited the lowest fluctuation values (1.0–1.4 Å), indicating a highly stable and rigid binding conformation. This observation supports the previously obtained docking results, where Voc showed the strongest binding affinity (− 11.6 kcal/mol), likely due to its extensive interaction network with the catalytic residues. Pim also exhibited low RMSF values (1.2–1.7 Å) around the active site, suggesting a robust and stable binding interface consistent with its high docking score and biological performance. In contrast, the AChE–Csa complex showed the greatest fluctuations (1.8–2.5 Å), suggesting a more dynamic binding interaction despite favorable docking energies. Tac displayed moderate stability, with RMSF values ranging from 1.6 to 2.0 Å, correlating with its intermediate docking performance. These MDS findings reinforce the docking predictions and experimental AChE inhibition data, particularly for Pim and Voc, which exhibited both high structural stability and functional potency. Thus, the integration of MD simulations strengthens the reliability of in silico predictions and further supports the potential of these CNIs as repurposed candidates for modulating cholinergic dysfunction in neurodegenerative diseases.

Voc formed the most extensive and geometrically favorable hydrogen bond network with AChE, including a strong short-range bond (1.99 Å) between its ligand H68 and the side-chain carboxyl group of GLU313. Several other interactions involved key residues such as ASN317 and GLY240, suggesting that Voc effectively stabilizes the binding pocket. This dense and well-oriented interaction pattern aligns with Voc’s superior performance in AChE inhibition assays and its pronounced neuroprotective effects observed in cellular experiments. Pim displayed multiple moderate interactions with the catalytic residue SER203, a critical residue within the oxyanion hole of AChE, with hydrogen bond distances between 2.90 and 3.14 Å. While these interactions support a stable binding, the observed bond angles and longer distances compared to Voc may account for its relatively lower AChE inhibition, despite showing potent anti-apoptotic properties. These findings reinforce the notion that Pim may exert neuroprotection through pathways beyond cholinergic modulation, such as inhibition of caspase activation, as confirmed by our caspase-3 data and supported by previous studies. Csa formed high-quality hydrogen bonds with GLU285 and LEU289, residues that lie close to the peripheral anionic site (PAS) of AChE. Although these bonds are strong and well-oriented (distances < 2.0 Å and DHA angles > 160°), their positioning outside the catalytic gorge may explain Csa’s modest inhibitory effect despite good binding affinity. Such peripheral binding could modulate enzyme conformation or allosteric dynamics without directly blocking the active site. Tac formed two weak hydrogen bonds (3.18 and 2.80 Å) with relatively poor angular geometry (DHA < 65°), suggesting a less stable and possibly transient interaction with the AChE binding site. This may underlie its lower efficacy in both inhibition and neuroprotection observed at the tested concentrations. Finally, the reference compound Gal exhibited two stable and geometrically favorable hydrogen bonds with LEU289 and GLN291 (2.38–2.47 Å, DHA > 140°), consistent with its established role as a potent reversible AChE inhibitor.

The ADMET results for Tac, Pim, Csa, and Voc provide valuable insights into their pharmacokinetic and toxicological profiles, which are critical for assessing their therapeutic potential in AD. The high molecular weights of these drugs, particularly Csa and Voc, may limit their oral bioavailability according to Lipinski’s Rule of Five, yet their calculated LogP values (ranging from 4.35 to 6.08) suggest strong Lipophilicity, which supports their ability to cross the BBB. The hydrogen bond donor and acceptor counts, alongside the TPSA values, indicate moderate permeability, balancing their ability to cross cellular membranes while maintaining solubility. Acute toxicity assessments reveal favorable profiles, as all compounds exhibit no signs of acute toxicity or mutagenicity. However, Pim displayed indications of genotoxic and non-genotoxic carcinogenicity, highlighting a potential safety concern that warrants further investigation. Interestingly, the PPB values, which exceed 80% for all drugs, suggest significant binding to circulating proteins. While this may reduce the free drug concentration available for immediate action, it could prolong their half-lives and therapeutic effects. The Vd values varied among the compounds, with Pim exhibiting the highest Vd (1.762), indicative of its extensive tissue distribution, which may influence its overall efficacy and safety. These ADMET profiles highlight the distinct pharmacokinetic and safety properties of each compound, while highlighting that all four drugs show potential for repurposing as AD therapies.

Although Pim exhibits a logP value exceeding 6.0—indicative of high lipophilicity and, by extension, greater membrane permeability and intracellular accumulation—this pharmacokinetic advantage did not translate into superior neuroprotective efficacy at the 0.1 μM concentration, as observed with other CNIs tested at the same dose. Notably, in our updated findings, Pim at 1 μM provided significantly greater protection against H_2_O_2_-induced cytotoxicity in SH-SY5Y cells compared to its 0.1 μM concentration. This result aligns with ADMET predictions, which suggest that higher lipophilicity enhances intracellular drug accumulation and may augment biological activity [89, 90]. However, this relationship appears to be both compound- and mechanism-specific. While Pim displayed superior cell viability at 1 μM in the MTT assay, it was less effective than Voc at inhibiting AChE activity at the same concentration. These findings suggest that the neuroprotective mechanism of Pim may be primarily anti-apoptotic rather than cholinergic, a conclusion supported by our recent study demonstrating the modulation of key apoptotic signaling pathways by 1 μM Pim in H_2_O_2_-exposed, neuron-like differentiated SH-SY5Y cells [22]. Collectively, these observations underscore that drug efficacy is a multifactorial outcome, influenced not only by physicochemical properties such as lipophilicity but also by compound-specific mechanisms of action and the cellular context in which the drug is evaluated.

The in vitro AChE inhibition results further underscore the potential of these CNI drugs as therapeutic agents for NDs. Voc demonstrated exceptional inhibitory activity at low concentrations, surpassing Gal at 0.01 µM and 0.1 µM. This finding suggests that Voc’s strong binding affinity, as revealed by molecular docking, translates effectively into functional inhibition of AChE activity. Voc is a CNI characterized by an improved calcineurin-binding profile and a reduced burden of drug and metabolites [91]. This unique pharmacokinetic-pharmacodynamic relationship allows for a high AChE inhibition profile at a dose associated with relatively low calcineurin inhibition. The higher concentrations of Voc (1 µM and 10 µM) exhibited a plateau or reduction in inhibitory efficacy, likely reflecting potential saturation effects or off-target interactions. In contrast, Tac, Pim, and Csa exhibited weaker in vitro inhibitory effects across all tested concentrations. While these drugs may not provide the same level of AChE inhibition as Voc, their moderate effects highlight the potential for their use in combination therapies or in targeting other pathways relevant to neuroprotection. The observed concentration-dependent effects also suggest that optimal dosing is critical for maximizing the therapeutic potential of these drugs while minimizing potential side effects.

Gal was included as a positive control in the in vitro AChE inhibition assays but was not assessed in the cellular neuroprotection experiments. However, this decision was based on the well-documented safety profile of Gal at concentrations up to 10 μM in SH-SY5Y cells. Previous studies have demonstrated that Gal exhibits negligible cytotoxicity at therapeutically relevant concentrations. For instance, Gal induced only modest toxicity (17% reduction in viability) at a concentration of 1.25 mg/mL, with a predicted IC_50_ of 3.53 mg/mL (12.2 mM) [92]. In a follow-up study, no toxic effects were observed at 0.1 μM, and significant reductions in viability occurred only at concentrations ≥ 100 μM [93]. Kola et al. [94] further confirmed that concentrations up to 50 μM had no cytotoxic effect on differentiated SH-SY5Y cells. Similarly, Girgin et al. [95] reported that Gal-induced cytotoxicity was concentration-dependent, but cell viability remained comparable to controls at concentrations ≤ 10 μM. In support of these findings, Wojtunik-Kulesza et al. [96] reported a CC_50_ value for Gal close to 5 mM, indicating a wide safety margin. These results collectively validate the concentration range (0–10 μM) used in our AChE assays and support the role of Gal as a reliable reference compound in neuropharmacological research.

The IC_50_ values derived from enzyme kinetics assays provide a quantitative evaluation of the AChE inhibitory potency of CNIs. Among the tested compounds, Voc exhibited a remarkably low IC_50_ value of 0.004 µM, indicating a strong inhibitory effect on AChE activity. In comparison, Tac (4.243 µM), Pim (2.148 µM), and CsA (2.335 µM) demonstrated weaker inhibition. Notably, Gal—a clinically approved AChEI—displayed an IC_50_ value of 0.212 µM in our study, which aligns closely with previously reported values using the Ellman method, typically ranging from 0.2 to 0.5 µM depending on experimental conditions and enzyme source [92, 97, 98]. This consistency not only validates the reliability of our assay but also reinforces the significance of Voc’s much lower IC_50_. The fact that Voc outperformed Gal in AChE inhibition suggests its high binding affinity and efficacy, possibly due to distinct structural interactions with the active site of the enzyme. Considering its additional neuroprotective effects observed in our cellular models, Voc emerges as a highly promising candidate for further investigation in the context of ND. These findings support the potential of CNIs, particularly Voc, as dual-function agents that may address both cholinergic deficits and oxidative stress–related neurotoxicity in diseases such as AD.

In addition to the effects observed with CNIs, it is important to consider established neuroprotective agents such as Gal for comparison. In addition to its well-known AChE inhibitory effects, Gal has been shown to exert direct neuroprotective actions in oxidative stress models. Zhang et al. [99] demonstrated that N-aryl galantamine analogues protected SH-SY5Y cells from H_2_O_2_-induced cytotoxicity. Also, Gal acts as an allosteric potentiating ligand of nicotinic acetylcholine receptors (nAChRs), especially the α7 and α3β4 subtypes, thereby enhancing cholinergic neurotransmission and promoting neuronal survival [100, 101]. Its neuroprotective effects involve activation of the α7 nAChR–phosphatidylinositol 3-kinase (PI3K)–protein kinase B (Akt) signaling pathway, stimulation of Aβ phagocytosis by microglia, and suppression of neuroinflammation through reduced production of pro-inflammatory cytokines (such as IL-6, IL-1β, and TNF-α) and inhibition of nuclear factor kappa B (NF-κB) signaling [102–104]. Gal also exhibits antioxidant properties by decreasing ROS levels and attenuating caspase-3 activation, which contributes to its anti-apoptotic activity. Additionally, it supports neuronal survival by upregulating neurotrophic factors such as nerve growth factor (NGF) and insulin-like growth factor 2 (IGF2) [105, 106]. Taken together, these diverse molecular actions distinguish Gal as a multifaceted neuroprotective agent. While our study focused on CNIs, which may act through distinct or partially overlapping mechanisms, future research directly comparing CNIs with Gal in oxidative stress models may help elucidate their relative therapeutic potential.

Building on this comparative framework, we next analyzed the dose-dependent cytotoxic and neuroprotective properties of CNIs under oxidative stress conditions in SH-SY5Y cells. The cytotoxicity and neuroprotective activity of the tested CNIs were further elucidated through a detailed analysis of their dose-dependent effects on SH-SY5Y cells. In the absence of oxidative stress (Fig. 3a), Tac and Voc at 1 and 10 μM concentrations significantly reduced cell viability, indicating potential cytotoxic effects at higher doses. In contrast, Pim and Csa did not show significant cytotoxicity at the tested concentrations, suggesting a more favorable safety profile under basal conditions. Our findings indicate that in (d)-SH-SY5Y cells subjected to oxidative stress via H_2_O_2_ treatment, cell viability, neurite length, and the number of neurites per cell were significantly reduced compared to untreated cells. This observation aligns with morphological and functional degeneration commonly reported in oxidative stress-related neurodegenerative conditions. These results are consistent with previous literature findings [23, 25, 107–109]. However, under oxidative stress conditions (Fig. 3b), simultaneous treatment with H_2_O_2_ and CNIs at 0.1 μM significantly attenuated H_2_O_2_-induced cytotoxicity. Interestingly, 1 μM Pim provided even greater protection than its 0.1 μM counterpart, an effect not observed with other CNIs. This may be attributed to the higher lipophilicity of Pim.

In contrast, 10 μM concentrations of Tac, Voc, and Csa when co-administered with H_2_O_2_ significantly reduced cell viability compared to H_2_O_2_ alone. This suggests that beyond a certain threshold, these compounds may exert synergistic toxicity with oxidative stress, potentially through mechanisms such as mitochondrial dysfunction, calcium overload, or increased ROS production.

Our findings underscore the differential effects of CNIs on oxidative stress-induced apoptosis in neuronal cells (Fig. 3d). The marked increase in caspase-3 levels following H_2_O_2_ exposure confirms the pro-apoptotic nature of oxidative damage. Notably, co-treatment with Pim and Voc significantly attenuated caspase-3 activation, indicating their potential to counteract H_2_O_2_-induced apoptotic signaling. While no prior studies have directly reported antioxidant properties for Voc, this observation is consistent with previously described antioxidant and cytoprotective effects of Pim [22]. In contrast, Tac and Csa did not exhibit comparable modulatory effects on caspase-3 levels under oxidative conditions, in agreement with existing literature [110, 111]. However, nanocarrier systems such as polyelectrolyte-coated nanocapsules have shown protective effects against oxidative damage in neuronal-like cells, with Csa-loaded formulations offering promising outcomes in oxidative stress models [25]. The ability of Pim and Voc to reduce apoptotic burden while preserving neuronal integrity supports their potential as neuroprotective agents in models of oxidative stress-associated neurodegeneration.

Among the tested compounds, Voc demonstrated the most pronounced preservation of neurite morphology, restoring both neurite length and density to levels comparable to those observed in untreated controls. This suggests that Voc not only reduces oxidative stress but also promotes cytoskeletal stability and neurite outgrowth, which are critical for maintaining neuronal connectivity and function.

The moderate effects observed with Tac, Pim, and Csa suggest that while these drugs confer some degree of neuroprotection, their mechanisms of action may not be as directly associated with cytoskeletal regulation or neurite extension as those of Voc. These findings support the hypothesis that Voc’s dual effects on both oxidative stress and neuronal morphology contribute to its superior neuroprotective profile. Furthermore, previous studies have demonstrated that CNIs can positively influence both cell survival and neurite morphology in continuous neuronal cell lines, microglial cells, and/or primary neuron cultures, further supporting their potential therapeutic applications [23, 68, 112].

In (d)-SH-SY5Y cells subjected to oxidative stress via H_2_O_2_ treatment, the increase in AChE activity underscores the enzyme’s involvement in oxidative stress and neurodegeneration. Previous data corroborate our findings, indicating that H_2_O_2_ treatment leads to an increase in AChE activity and a decrease in cell viability in SH-SY5Y cells [113, 114]. According to our results, Voc is the drug that best prevents AChE inhibition caused by H_2_O_2_. Tac, Pim, and Csa also demonstrated AChE inhibitory effects, albeit to a lesser extent than Voc. Their relatively lower efficacy in reducing AChE activity may be explained by differences in their structural interactions with the enzyme.

Detailed analyses, including qRT-PCR, confirmed that Voc significantly downregulated AChE mRNA levels, thereby supporting its role in modulating AChE expression. Tac, Pim, and Csa also exhibited a reduction in AChE activity, although their effects were less marked compared to Voc. Despite their relatively lower potency in AChE inhibition, these inhibitors still present valuable therapeutic potential due to their neuroprotective effects. Previous studies have reported that Tac and Csa reduce AChE mRNA levels in HeLa cells [115] and that Csa decreases AChE mRNA stability in C2–C12 muscle cells [116]. Additionally, Kumar and Singh [60] investigated the effects of CNIs on a mouse model of dementia and reported positive outcomes, particularly in mitigating memory loss and neuropathological changes. Their findings demonstrated that Tac and Csa significantly reduced AChE activity in the brains of treated mice. This reduction was accompanied by an increase in ACh levels, leading to improved memory performance and enhanced neurological health [60]. However, there is no literature available regarding the AChE inhibitory effects of Voc. This study is the first to explore this aspect. Although previous studies demonstrating the pharmacological properties of Voc did not mention its direct effects on AChE [117], it may indirectly influence AChE activity through its effects on the immune system, regulation of inflammatory processes, and modulation of calcium signaling. Voc’s superior AChE inhibitory activity compared to other CNIs may be attributed to its enhanced pharmacokinetic properties and optimized structural interactions with calcineurin and related pathways. Unlike Tac, Pim, and Csa, Voc is a next-generation CNI that has been chemically modified to exhibit increased binding affinity and specificity to calcineurin [91, 118, 119]. Studies have shown that structural modifications in Voc improve its lipophilicity and BBB penetration compared to earlier CNIs, which may further contribute to its neuroprotective effects by ensuring effective central nervous system (CNS) delivery [4]. This higher specificity may facilitate more precise modulation of calcineurin-dependent pathways, which are implicated in calcium dysregulation and cholinergic dysfunction, two critical aspects of AD pathology. Furthermore, calcineurin inhibition can downregulate the overexpression of AChE induced by oxidative stress, as calcineurin activity has been linked to the transcriptional regulation of stress-responsive genes, including those affecting cholinergic signaling [120]. Collectively, these attributes make Voc a promising candidate for further exploration as a dual-targeted therapy, addressing both neuroinflammation and cholinergic dysfunction in AD.

While the present findings highlight the neuroprotective and AChE inhibitory effects of CNIs, a deeper understanding of their underlying mechanisms beyond immunosuppression is crucial to fully appreciate their therapeutic potential. Therefore, it is important to briefly discuss the established pathways through which CNIs may exert direct neuroprotective actions and influence AChE regulation.

In addition to their well-established immunosuppressive properties, CNIs have emerged as promising neuroprotective agents, acting through diverse mechanisms beyond T-cell suppression. CNIs modulate several critical pathways in neuronal cells, including calcium homeostasis, oxidative stress responses, mitochondrial integrity, and apoptotic signaling. They exert anti-apoptotic effects by stabilizing mitochondrial membranes, inhibiting the mitochondrial permeability transition pore (MPTP), and preventing the release of cytochrome c. Notably, Tac has been shown to block calcineurin-mediated dephosphorylation of the pro-apoptotic protein Bad, thereby reducing neuronal apoptosis and promoting survival under oxidative stress conditions [121–123].

Beyond apoptosis regulation, CNIs interact with signaling cascades such as ERK1/2, JNK, and p38 MAPK, contributing to their anti-inflammatory and neuroprotective profiles. For instance, Tac has been shown to inhibit MAPK phosphorylation in LPS-treated microglial cells, reducing the expression of inflammatory mediators like TNF-α and IL-6 and thereby mitigating glial reactivity [124, 125]. In addition, CNIs may indirectly enhance autophagy and promote BDNF trafficking, both essential for neuronal maintenance and synaptic plasticity [21, 126].

Regarding AChE regulation, CNIs influence this enzyme at both transcriptional and post-transcriptional levels. Evidence suggests that calcineurin inhibition stabilizes AChE mRNA, possibly by interfering with calcineurin-sensitive regulatory proteins that control mRNA degradation [116, 127]. Interestingly, studies have shown that while CsA enhances AChE expression in skeletal muscle by increasing mRNA stability, Tac may exert similar effects in neuronal tissues, albeit via different mechanisms involving the FKBP-12 immunophilin complex [128, 129]. These effects may vary across tissues, highlighting the importance of context-dependent regulation.

Furthermore, CNIs modulate intracellular calcium and oxidative signaling, both of which are known to influence AChE expression. Elevated calcium levels and oxidative stress can upregulate AChE, exacerbating cholinergic dysfunction. By dampening these stressors, CNIs may normalize AChE activity and restore cholinergic tone. Additionally, there is emerging evidence that CNIs may interfere with transcriptional regulators such as NFAT and CREB, which participate in AChE gene regulation under stress conditions [30, 130].

These multifaceted mechanisms suggest that CNIs not only complement classical AChE inhibitors but also offer broader therapeutic potential by targeting upstream molecular stressors that drive cholinergic dysfunction. However, the paradoxical upregulation of AChE transcripts in some tissues following CNI treatment necessitates a careful evaluation of tissue specificity, dosage, and context when considering clinical applications in ND.

Given the dual role of these drugs in modulating AChE activity and providing neuroprotection, they represent promising candidates for further development as therapeutic agents for NDs, particularly AD. The repurposing of immunosuppressive agents offers several advantages, including well-established safety profiles and pharmacokinetic data, which could expedite their clinical development for AD and other NDs. Future studies should focus on elucidating the precise molecular mechanisms underlying their neuroprotective effects, as well as conducting in vivo experiments to validate their therapeutic potential. The integration of these drugs into the therapeutic landscape could address some of the limitations of current AChE inhibitors, such as variable efficacy and adverse side effects, ultimately improving outcomes for patients with AD.

## Conclusion

In conclusion, our study highlights the potential of CNIs as novel therapeutic agents for NDs characterized by cholinergic dysfunction and oxidative stress. Among the tested compounds, Voc emerged as particularly promising due to its substantial neuroprotective effects and notable reduction in AChE activity in neuron-like SH-SY5Y cells exposed to oxidative stress. Although Tac, Pim, and Csa also exhibited beneficial effects, their impact was less pronounced. These findings support further investigation into the potential therapeutic applications of CNIs, particularly Voc, in the treatment of NDs.

## Limitations and Future Perspectives

Despite the promising in vitro findings regarding the neuroprotective and AChE-inhibitory effects of CNIs, several limitations must be acknowledged. Most notably, CNIs such as Tac, CsA, and Pim are known to exert systemic immunosuppressive effects and have been associated with adverse outcomes including nephrotoxicity, neurotoxicity, and increased susceptibility to infections in long-term clinical use [131]. These potential off-target effects could pose significant challenges for their repurposing in chronic neurological conditions such as AD.

Furthermore, the BBB permeability of CNIs is a critical consideration for their effective use in CNS disorders. Accumulating evidence suggests that CsA and Tac can increase BBB permeability through multiple mechanisms, including inhibition of the efflux transporter P-glycoprotein (P-gp) and induction of apoptosis in brain capillary endothelial cells. These actions disrupt the integrity of the BBB and allow enhanced accumulation of substances within the brain [132, 133]. While this may facilitate CNS drug delivery, it may also lead to undesirable neurological effects, particularly during prolonged exposure.

In contrast, Pim and Voc, due to their higher molecular weights (810 Da and 1215 Da, respectively) and limited systemic use (particularly in the case of Pim), are less likely to cross the BBB via passive diffusion. To date, there is no conclusive evidence of Voc interacting with P-gp or disrupting endothelial cell integrity. Its favorable CNS safety profile in clinical settings further suggests minimal BBB permeability, although dedicated pharmacokinetic and CNS distribution studies are needed to confirm this.

In addition to caspase-3 activation, which we evaluated in this study as an apoptosis-related marker, future work should explore other oxidative stress biomarkers such as intracellular ROS accumulation, lipid peroxidation, and antioxidant enzyme activity. These markers would allow for a more comprehensive understanding of the redox-mediated neuroprotective mechanisms of CNIs under oxidative injury. Furthermore, caspase-3 ELISA experiments were conducted with two biological replicates. While the observed trends—particularly the significant reduction by Pim and Voc—were consistent and reproducible across these replicates, the limited sample size necessitates cautious interpretation. Additional replicates will be required in future studies to validate these findings.

Furthermore, while our findings demonstrate acute neuroprotection, the long-term effects of CNIs on neuronal health and synaptic function remain to be determined. Chronic neurodegenerative conditions involve progressive synaptic loss and network dysfunction; therefore, assessing parameters such as neurite complexity, synaptic protein expression, and electrophysiological activity in extended models will be essential for establishing sustained therapeutic benefits.

Additionally, although our study employed a concentration (0.1 µM) selected based on initial cytotoxicity screening, we acknowledge the need for a dose–response analysis to better delineate the therapeutic window. CNIs are known to exhibit cytotoxicity at higher concentrations through mechanisms involving mitochondrial dysfunction and oxidative damage [134, 135]. A detailed dose–toxicity evaluation will be necessary to balance efficacy and safety in potential translational applications.

Moreover, one important limitation of the present study is the exclusive use of a single cellular model ((d)-SH-SY5Y cells). While this model provides valuable insights into neuronal responses under oxidative stress, it may not fully recapitulate the complexity of in vivo neuronal environments. Validation of findings across additional neuronal or glial cell lines, as well as primary cultures, would strengthen the generalizability and translational relevance of the results.

Future studies should aim to validate the current in vitro findings in relevant in vivo models of ND. Such investigations are necessary to evaluate long-term therapeutic efficacy, drug bioavailability in the brain, behavioral outcomes, and systemic and neurological safety. Furthermore, it would be valuable to explore targeted delivery strategies and modified formulations of CNIs that enhance CNS selectivity while minimizing systemic immunosuppressive effects.

## Supplementary Information

Below is the link to the electronic supplementary material.

ESM 1 (TIF 1.39 MB)

ESM 2 (TIF 1.68 MB)

ESM 3 (DOCX 14.4 KB)

## Data Availability

Data will be provided by the author upon request.
