# Supplementary figures and images for: Evaluating the Neuroprotective and Acetylcholinesterase Inhibitory Properties of Four Calcineurin Inhibitor Drugs: Tacrolimus, Pimecrolimus, Cyclosporin A, and Voclosporin
Source: Mol Neurobiol. 2025 Sep 15;62(12):16592–616. doi: 10.1007/s12035-025-05149-0 (PMC12559140; doi:10.1007/s12035-025-05149-0)

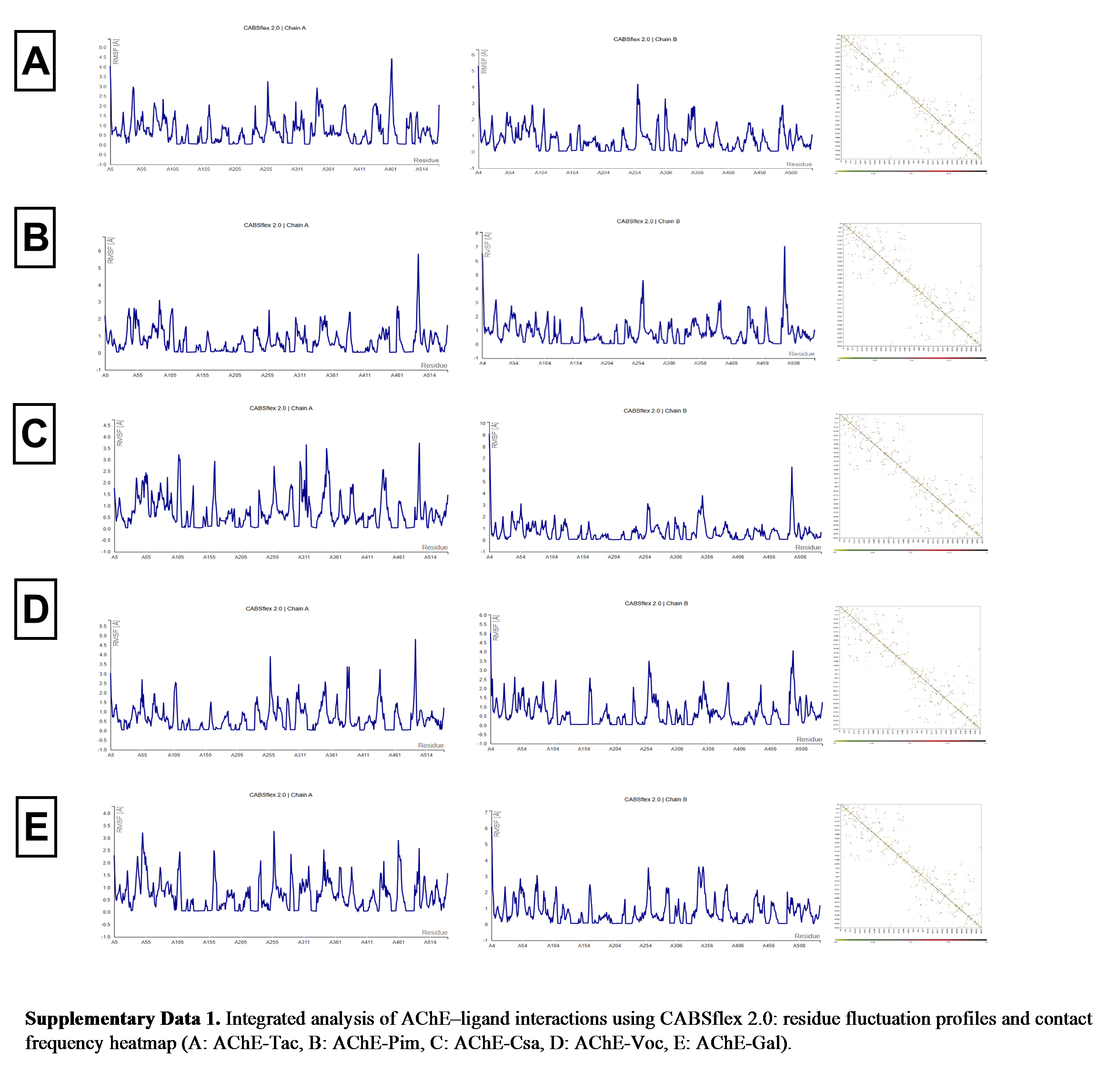

Supplement: Supplementary file 1 — (TIF 1.39 MB) [file 12035_2025_5149_MOESM1_ESM.tif]

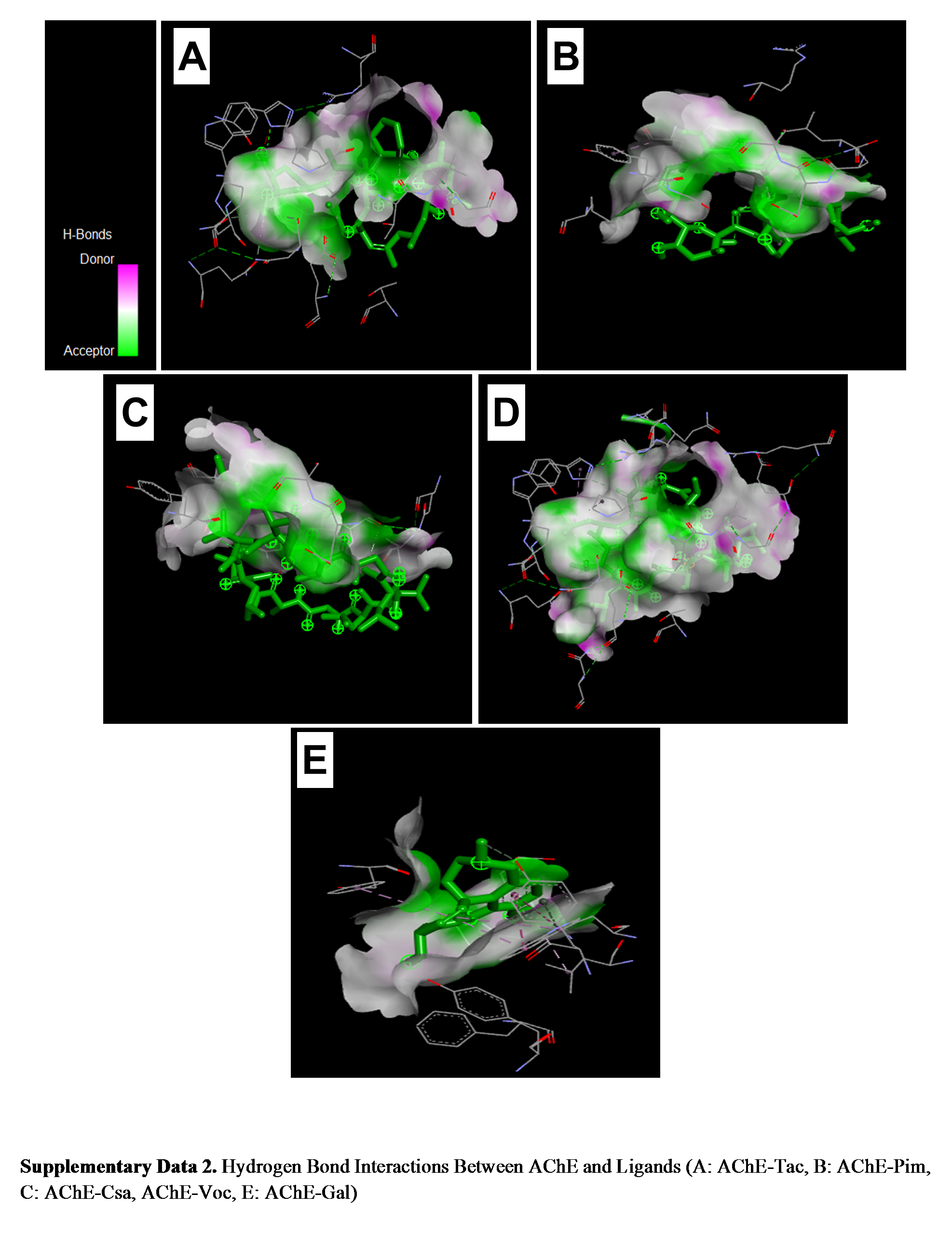

Supplement: Supplementary file 2 — (TIF 1.68 MB) [file 12035_2025_5149_MOESM2_ESM.tif]
