# Supplementary material for: Evaluating the Neuroprotective and Acetylcholinesterase Inhibitory Properties of Four Calcineurin Inhibitor Drugs: Tacrolimus, Pimecrolimus, Cyclosporin A, and Voclosporin
Source: Mol Neurobiol. 2025 Sep 15;62(12):16592–616. doi: 10.1007/s12035-025-05149-0 (PMC12559140; doi:10.1007/s12035-025-05149-0)
Supplement: Supplementary file 3 — (DOCX 14.4 KB) [file 12035_2025_5149_MOESM3_ESM.docx]

## Supplementary Data 3. Hydrogen Bond Interactions Between AChE and Ligands as Determined by Discovery Studio Visualizer

| **Complex** |  | **Donor Residue (Atom)** |  | **Acceptor Residue (Atom)** |  | **Distance (Å)** |  | **DHA Angle (°)** |  | **HAY Angle (°)** |
| --- | --- | --- | --- | --- | --- | --- | --- | --- | --- | --- |
| AChE–Tac |  | HIS405:ND1 |  | UNL1:O1 |  | 2.80 |  | 37.48 |  | 43.52 |
|  |  | ASN533:N |  | ASN533:OD1 |  | 3.18 |  | 64.19 |  | 70.90 |
| AChE–Pim |  | GLY121:N |  | UNL1:O |  | 3.11 |  | 45.56 |  | 10.46 |
|  |  | TYR124:OH |  | UNL1:O |  | 2.95 |  | 99.35 |  | 62.97 |
|  |  | SER203:OG |  | UNL1:O |  | 2.90 |  | 110.33 |  | 95.72 |
|  |  | SER203:OG |  | UNL1:O |  | 3.14 |  | 85.37 |  | 82.51 |
|  |  | ALA204:N |  | SER203:OG |  | 2.59 |  | 35.61 |  | 31.01 |
|  |  | TYR341:N |  | PHE338:O |  | 3.17 |  | 16.11 |  | 118.89 |
|  |  | TYR341:OH |  | ASP74:OD2 |  | 3.01 |  | 35.08 |  | 102.01 |
|  |  | UNL1:H |  | UNL1:O |  | 2.48 |  | — |  | — |
| AChE–Csa |  | GLN279:HE21 |  | ASN283:OD1 |  | 1.78 |  | 158.08 |  | 124.55 |
|  |  | GLU285:HN |  | VAL282:O |  | 1.97 |  | 165.17 |  | 107.05 |
|  |  | LEU289:HN |  | TRP286:O |  | 2.44 |  | 169.92 |  | 116.30 |
| AChE–Voc |  | VAL239:N |  | PRO301:O |  | 2.90 |  | 21.48 |  | 158.46 |
|  |  | GLY240:N |  | UNL1:O5 |  | 3.16 |  | 26.78 |  | 102.98 |
|  |  | ARG296:NH2 |  | HIS405:NE2 |  | 3.07 |  | 23.75 |  | 115.09 |
|  |  | VAL303:N |  | VAL239:O |  | 3.14 |  | 11.67 |  | 175.10 |
|  |  | GLU313:N |  | GLU313:OE2 |  | 2.86 |  | 26.54 |  | 105.15 |
|  |  | ASN317:ND2 |  | GLU313:O |  | 3.14 |  | 81.68 |  | 128.78 |
|  |  | ASN533:N |  | ASN533:OD1 |  | 3.18 |  | 64.19 |  | 70.90 |
|  |  | LEU540:N |  | PRO537:O |  | 3.21 |  | 48.58 |  | 107.76 |
|  |  | SER541:N |  | PRO537:O |  | 3.07 |  | 29.80 |  | 156.14 |
|  |  | UNL1:H68 |  | GLU313:OE1 |  | 1.99 |  | — |  | — |
| AChE–Gal |  | LEU289:HN |  | TRP286:O |  | 2.38 |  | 163.01 |  | 111.67 |
|  |  | SER293:HG |  | GLN291:O |  | 2.47 |  | 141.06 |  | 146.55 |

Note: Hydrogen bond distances and angles were determined using Discovery Studio Visualizer. DHA: Donor–Hydrogen–Acceptor angle; HAY: Hydrogen–Acceptor–Y angle. “—” denotes missing or not measurable values.
